# Autonomous Switching of Self-Propelled Motion Modes of Hinokitiol-Fueled Elastomer Matrices

**DOI:** 10.1021/jacs.5c17346

**Published:** 2025-10-20

**Authors:** Lara Rae Holstein, Masayuki Takeuchi, Nobuhiko J. Suematsu, Atsuro Takai

**Affiliations:** † Molecular Design and Function Group, 52747 National Institute for Materials Science (NIMS), 1-2-1 Sengen, Tsukuba, Ibaraki 305-0047, Japan; ‡ Department of Materials Science and Engineering, Faculty of Pure and Applied Sciences, University of Tsukuba, 1-1-1 Tennodai, Tsukuba, Ibaraki 305-8577, Japan; § School of Interdisciplinary Mathematical Sciences; Graduate School of Advanced Mathematical Sciences; Meiji Institute for Advanced Study of Mathematical Sciences (MIMS), Meiji University, 4-21-1, Nakano, Tokyo 164-8525, Japan

## Abstract

Programmable self-propelled motion underpins essential biological functions and serves as a powerful inspiration in the design of dynamic synthetic materials. While significant progress has been made in developing self-propelled systems, most existing strategies rely on external stimuli or the incorporation of coupled oscillatory chemical reactions to achieve mode switching. In contrast, approaches that enable intrinsic switching between motion modes—such as from continuous to oscillatory—without external control remain limited. In this study, we introduce a self-propelled disk utilizing hinokitiol as a surface-active “fuel” within a polystyrene elastomer matrix, floating on the water surface. Hinokitiol-containing disks exhibited spontaneous transitions from continuous to oscillatory movement, distinctly without the need for external inputs. By leveraging the phase transitions of hinokitiol and tuning the mesoscale structure of the polymer scaffold, we succeeded in modulating the duration of continuous motion and frequency of oscillation in the macroscopic motion of the disks. These findings demonstrate that life-like macroscopic motion can be systematically engineered by coordinating the molecular arrangement of fuel species and the mesoscale structures of the surrounding polymer scaffold, presenting a versatile molecular design approach for synthetic self-propelled materials.

## Graphical abstract

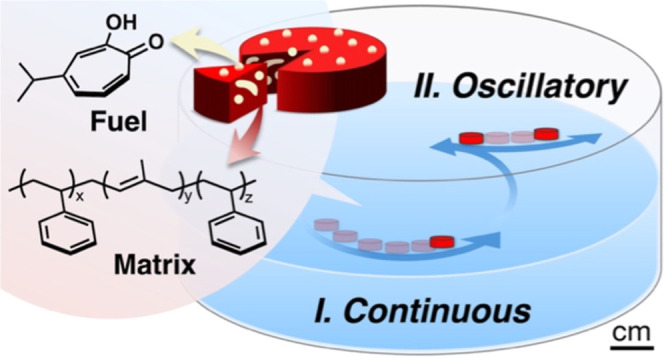

## Introduction

Autonomous, periodic motion underlies a variety of sophisticated functionalities in living organisms, such as cardiac beat and peristaltic motion. Emulating such nonlinear behavior in synthetic systems remains a formidable challenge, but offers exciting opportunities for the development of new soft materials capable of self-sustained dynamic and adaptive responses.^1–5^ These advances could greatly benefit potential applications in soft robotics, medicine, and materials science.^6–11^ A number of synthetic systems have been reported to undergo self-propelled motion,^12–29^ typically exemplified by solid disks floating on water surfaces and oil droplets in aqueous solution, which are driven by the release of surface-active “fuel” molecules that generate interfacial surface tension gradients known as Marangoni flow.^27,30–37^ Some of these systems exhibit oscillatory motion by implementing inherently oscillatory reactions, such as enzymatic reaction cycles^38–40^ and the Belousov–Zhabotinsky reaction,^41–44^ or via chemical reactions that alter physicochemical parameters,^45–48^ such as surface tension, which govern self-propelled motion. Oscillatory motion is also known to occur in response to the environmental conditions around the self-propelled objects, such as the density of surfactants at the interface.^49–55^ In these aforementioned examples, the mode of motion remains unchanged unless environmental changes or additional species are deliberately introduced. Therefore, methods for inducing transitions over time between different modes of motion–for instance from continuous to oscillatory motion–within a single system without external inputs have largely been overlooked.

There are a few examples of switching modes of motion in systems where Marangoni fuels are released from a scaffolding material, such as metal–organic framework (MOF) and agarose gel.^56–61^ These reports suggest the mode switching may arise from the modulated diffusion of the fuel through the scaffolding matrix. Although diffusion and release of small molecules from MOFs^62–64^ and various polymer matrices^65–67^ have been extensively studied, the potential for controlling mode switching behavior of self-propelled systems has not been explored. We envisioned that tunable macroscopic motion could be achieved by considering the polymer mesoscale structure in combination with the physiochemical properties of the chosen fuel species.

Here we present a simple system that demonstrates fully autonomous switching between continuous and oscillatory modes of motion, achieved solely through the solid–liquid phase transition of a surface-active fuel and the phase-separated structures of the polymer matrix without any external inputs. We employed hinokitiol (**HT**) as the fuel species and polystyrene elastomer as the polymer scaffold (Figure 1). **HT**, a member of the tropolone family, is an aromatic compound with a seven-membered ring structure. Since its isolation from the wood of *chamaecyparis taiwanensis* in the 1930s,^68^ **HT** has garnered attention not only for its unusual structural chemistry,^69–72^ but also for its pharmacological activity in the fields of medicine and consumer products.^73–76^ Meanwhile, polystyrene elastomer is a widely used copolymer known to exhibit various phase-separated structures depending on its composition.^77^ We found that, when placed on water, disks composed of **HT** blended with an elastomer scaffold showed sequential mode switching from continuous to oscillatory mode without external input (Figure 1). Notably, the motility of the disk on the centimeter scale can be controlled by simply altering the disk fabrication procedure or by varying the composition of the polymer matrix. We systematically investigated the underlying mechanisms of this phenomenon using various fuel species and polymer matrices.

**Figure 1:**
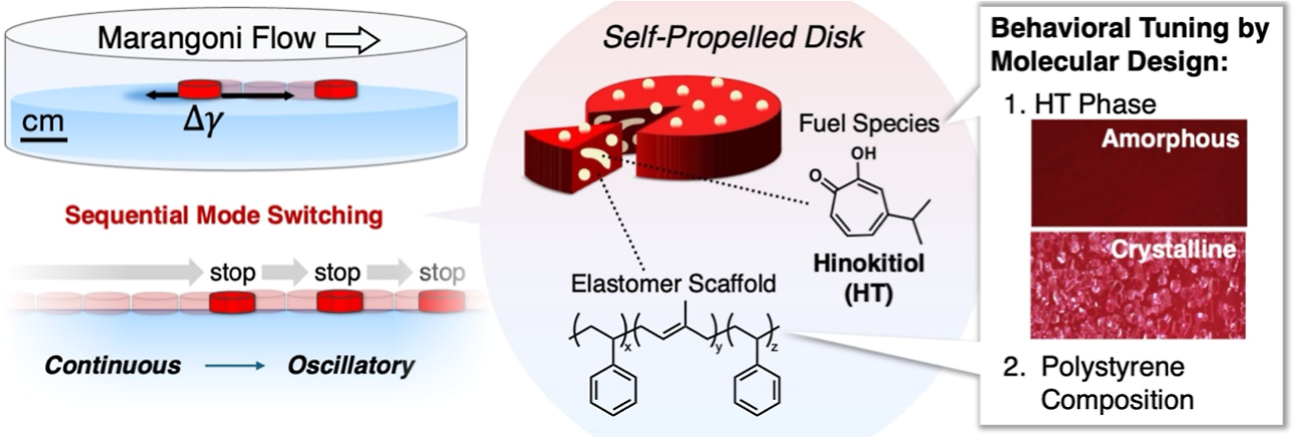
Chemical structures and schematic illustration of spontaneous switching from continuous to oscillatory motion caused by **HT** which can be tuned by adjusting the phase of **HT** and molecular composition of the disk scaffold.

## Results and Discussion

### Sequential Mode Switching of HT-Elastomer Disks

When floated on distilled water at pH 7 under ambient conditions in a Petri dish, a small clump of **HT** crystals exhibited random, continuous motion consisting of large and small loops and twirling patterns (Figure 2a and Supporting Information Movie 1). The motion endured until the clump was spent, lasting up to ca. 2 h depending on the amount of sample. Such long-lasting behavior suggests **HT** does not accumulate on the surface of water, but rather is removed via dissolution into bulk solution. Although **HT** exhibits sublimation properties, its mass loss over 300 min is only about 7% (Figure S1), indicating that the contribution of sublimation to the overall removal process is minor. The self-propelled motion of the **HT** clump is comparable to that of the long-studied camphor,^78,79^ which is driven by the formation of a surface tension gradient that generates Marangoni flow.

**Figure 2:**
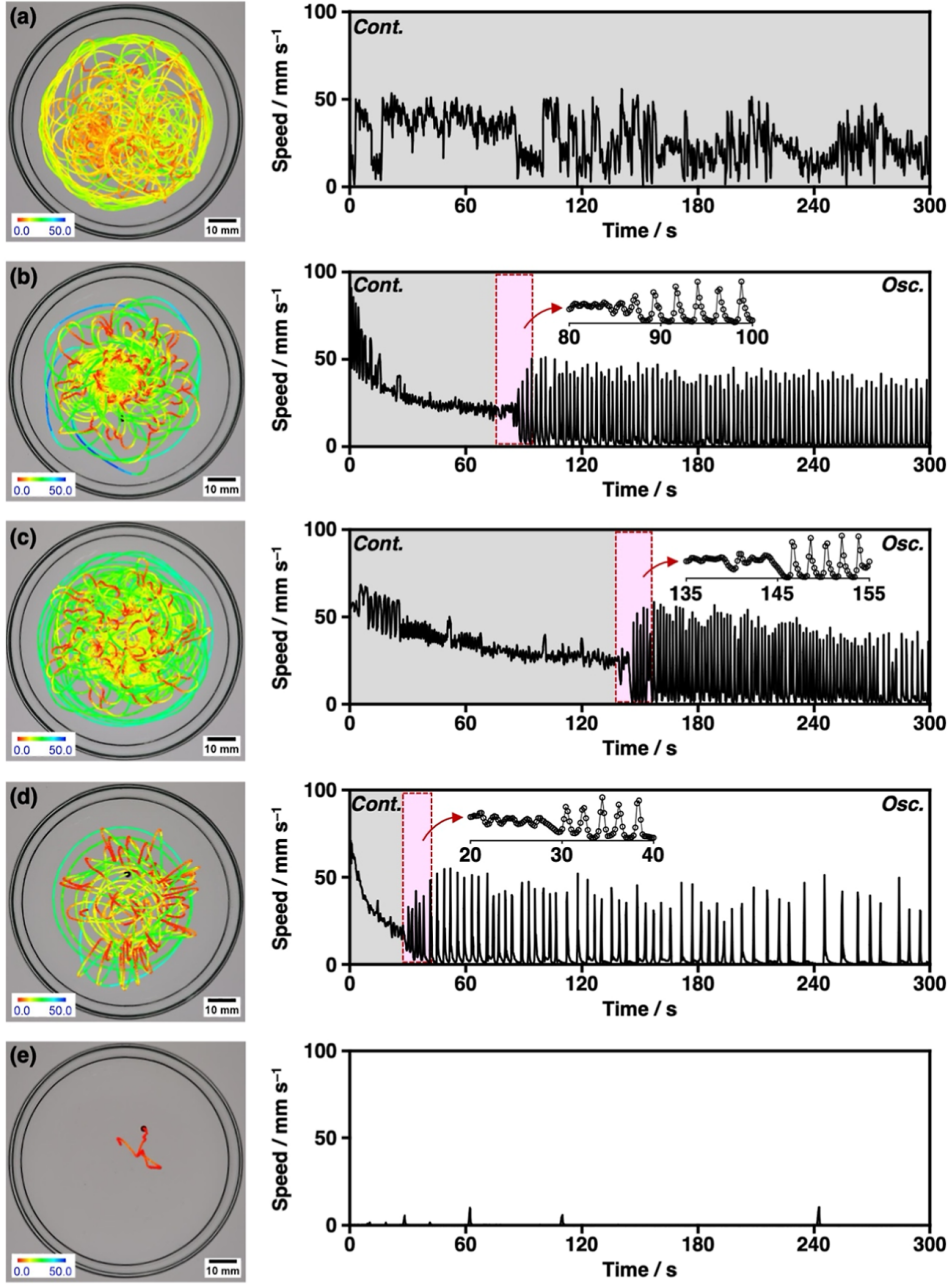
Motion fueled by **HT**. Trajectory and speed profiles of (a) a crystalline **HT** clump, (b) **HT-SIS**, (c) **cHT-SIS**, (d) **HT-SIS50**, and (e) **HT-PS** disks floated on distilled water at 25 °C. The gray regions indicate the continuous motion phase, while the white regions indicate the oscillatory motion phase. Enlarged views of the switching regions (red highlights) are shown in the insets.

A remarkable degree of sophistication emerged when disks were fabricated by combining **HT** with an elastomer scaffold. We prepared sheets of **HT** blended with an elastomer, polystyrene-polyisoprene-polystyrene triblock copolymer with 22 wt % styrene (**SIS**), in a 1:4 weight ratio which were subsequently cut into 2 mm diameter disks of 0.5 mm thickness, henceforth referred to as **HT-SIS** (see Supporting Information for the preparation methods). Initially, disks moved in rapid, continuous circles before transitioning via several small oscillations into an oscillatory mode characterized by periodic alternations between rest and rapid jumping which produced flower-like trajectory patterns (Figure 2b and Supporting Information Movie 2). Upon closer inspection of the **HT-SIS** disk, we observed that during the pauses in the oscillatory mode, the disk rotates in place (Supporting Information Movie 2, from 1:00 to 1:25). This rotation can rationally explain why the subsequent motion alternates back and forth rather than continuing in the same direction, as it perturbs the flow symmetry at the water surface. The duration of the continuous mode was on average 70 ± 10 s and the oscillatory motion continued for ca. 6.5 h, with a gradual decrease in frequency. The average speed of the rhythmic jumping was 40 ± 3 mm s^–1^ with a frequency of 0.43 ± 0.09 s^–1^ in the first 300 s. The friction coefficient of the disk on water can be calculated from the relaxation process during one jump cycle in the oscillatory mode. The frictional coefficient obtained from the first five jumps was determined to be 3.46 (±0.13) × 10^–6^ N m^–1^ s (see Figure S2 for details), which is comparable to that of a plastic boat equipped with camphor.^79^

To investigate the source of this mode switching, we allowed a fresh disk to swim for 300 s before relocating it to a second Petri dish with fresh water (Figure S3). The used disk immediately started oscillating, while a fresh disk added to the first Petri dish moved continuously before it started oscillating. Continuous to oscillatory type mode switching was still observed when the water depth was increased from 3 mm to 6 mm, and when the Petri dish diameter was changed from 75 mm to 50 mm, indicating the behavioral transition is not induced by the physical confines of the aqueous phase. When the surface of a **SIS** disk was coated with **HT**, only the continuous mode was consistently observed, and the duration of motion varied with the amount of **HT**; meanwhile, the oscillations became less frequent or disappeared entirely (Figure S4a and Supporting Information Movie 3). Disks constructed of just the **SIS** elastomer and the visualizing dye did not show self-propelled motion (Figure S4b and Supporting Information Movie 4). These results indicate that a layer of **HT** on the outer surface of the disk is responsible for the initial continuous mode, while **HT** inside the **SIS** elastomer disk may be responsible for the subsequent oscillatory mode.

### Control of Motion Mode Using Phase Change of HT

The phase-changing capability of **HT** provides an opportunity to alter its diffusion rate through the polymer scaffold. When a freshly prepared **HT-SIS** sheet was kept in a cold bath at −60 °C for 10 min and returned to ambient temperature, the elastomer sheet changed from opaque red to a dusty pink (Figure 3a). The cold-treated sheet, designated **cHT-SIS**, was also markedly stiff in contrast to the soft, slightly sticky **HT-SIS**. When placed on water at 25 °C, **cHT-SIS** disks displayed a longer continuous mode and higher frequency oscillations than **HT-SIS** disks (Figure 2c and Supporting Information Movie 5). The average duration of the continuous mode for **cHT-SIS** increased to 151 ± 36 s and the frequency of the oscillatory mode was 0.59 ± 0.14 s^–1^ with an average jump speed of 44 ± 8 mms^–1^. The self-propelled behaviors of **HT** disks with various compositions are summarized in Table 1.

**Figure 3:**
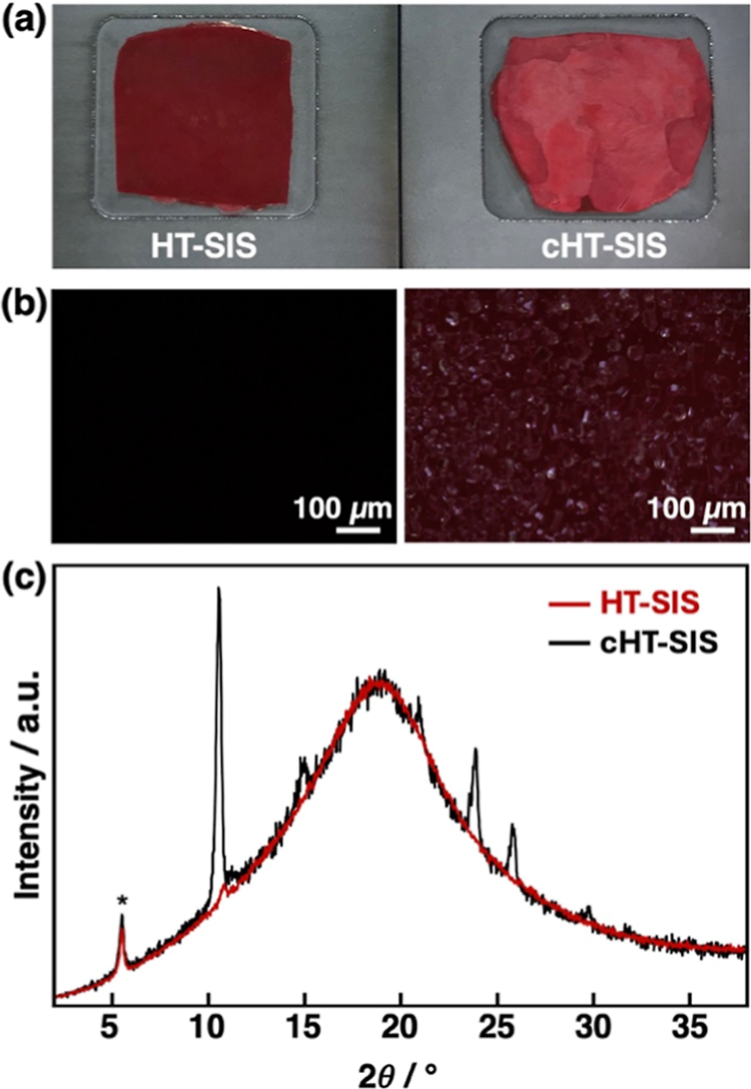
(a) Pictures of **HT-SIS** (left) and **cHT-SIS** (right) sheets. (b) Optical textures of **HT-SIS** (left) and **cHT-SIS** (right) observed under POM. (c) XRD patterns of **HT-SIS** (red line) and **cHT-SIS** (black line). The asterisk at 2θ = 5.6° (1.60 nm) denotes the peak corresponding to visualizing dye.

**Table 1:** Summary of the Self-Propelled Behavior of Different HT-Polymer Disks When Floated on Distilled Water Under Ambient Conditions^a^

disk name	duration of continuous motion/s	frequency of oscillatory motion/s^–1^	speed of jumping/mm s^–1^
**HT-SIS**	70 ± 10	0.43 ± 0.09	40 ± 3
**cHT-SIS**	151 ± 36	0.59 ± 0.14	44 ± 8
**HT-SIS50**	45 ± 14	0.28 ± 0.09	32 ± 5
**HT-PS**		– (irregular)	8 ± 9

a Experiments were conducted at least 6 times using fresh materials. The values were calculated for the first 300 s, along with their standard deviations.

To verify the tunability of the physical state of **HT**, we observed the melting of **HT** crystals to an isotropic oil at 52 °C. The sample remained in this liquid state long after it was cooled to 25 °C, demonstrating that **HT** can exist in both isotropic liquid and crystalline solid states at room temperature (Figure S5a). This supercooling phenomenon was also confirmed by differential scanning calorimetry (DSC) of pristine **HT** (Figure S5b). The elastomer itself may undergo a glass transition of its isoprene domain during the cold bath submersion, but otherwise did not show any changes within the temperature range of the sheet preparation conditions (Figure S5c). Observation under a polarized optical microscope (POM) revealed the surface of **cHT-SIS** was covered in polycrystals; no such optical textures were observed on the surface of **HT-SIS** (Figure 3b). As mentioned above, **HT-SIS** was molded by pressing at the melting point of **HT**, and **cHT-SIS** was prepared by subsequent cooling at −60 °C. Considering these preparation methods, the **HT-SIS** disk consists of fluid, amorphous **HT**, while the **cHT-SIS** disk primarily consists of crystalline counterparts. X-ray diffraction (XRD) measurements confirmed the increased crystallinity of **cHT-SIS** (Figure 3c). The peak pattern is consistent with the simulated XRD pattern from single crystal X-ray structural analysis of **HT** (Figure S6a). By contrast, the broad diffraction of **HT-SIS** indicates amorphous **HT** as the main component (Figure S6b). Thus, the duration time of the continuous motion and the frequency of the oscillating motion can be controlled by the amorphous and crystalline states of **HT**, as shown in Figure 2b,c.

To explain the causality between disk behavior and the crystallinity of **HT**, we considered how the latter affects the dissolution and supply from the disk onto the surface of the surrounding water. Time-dependent UV–vis absorption spectroscopy was employed to monitor the dissolution rate of **HT** from **HT-SIS** and **cHT-SIS** disks (Figure S7). A disk of either **HT-SIS** or **cHT-SIS** was placed on the surface of water in a cuvette, and the time profiles of the absorbance at 345 nm due to **HT** was monitored. The dissolution rate constant and final concentration of released **HT** were calculated by fitting to the Noyes–Whitney equation (see the Supporting Information for details). The resulting values are summarized in Table S1. These results suggest **HT** deeper within the disk can more easily diffuse when in the amorphous state (**HT-SIS**).

As a consequence of the dampened supply of **HT** from its crystalline form, **cHT-SIS** showed a more pronounced change in behavior when placed on cold water compared to **HT-SIS** (Figure S8). Therefore, we floated the **cHT-SIS** disk on water at different temperatures and obtained the speed profile as shown in Figure 4. Initially, when the disk was floated on water at 25 °C, mode switching from continuous to oscillatory motion was observed at 98 s. When the disk was transferred to water at 5 °C, the frequency of oscillatory motion immediately decreased probably due to the slow diffusion of **HT**.^80^ Upon returning the disk to water at 25 °C, both the frequency and speed of oscillatory jumping increased, approaching their original values. These observations indicate that **cHT-SIS** can switch its motion modes not only based on its own temperature history, but also in response to changes in the surrounding temperature.

**Figure 4:**
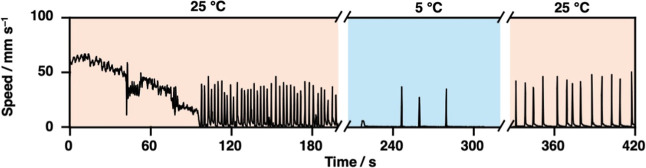
Speed profiles of **cHT-SIS** disks floated on distilled water at different temperatures. The time taken to transfer the disks to different Petri dishes is not shown.

### Mechanism of Motion Mode Switching

The oscillatory mode of **HT-SIS** does not require coupling to a chemical reaction or additional surfactants. Such spontaneous transition of motion mode has been reported to show slight intermittent motion just before cessation when camphoric acid mixed with agarose gel is used.^58^ However, the oscillatory motion of **HT-SIS** continued at a speed exceeding the average speed of continuous motion. Figure 5a illustrates the concentration profiles of **HT** released from the disk at different behavioral stages. We surmised the continuous mode arises from an initial layer of **HT** at the disk–water interface that is readily supplied to the local water surface. Note that the surface tension of aqueous solutions containing **HT** decreased linearly with increasing **HT** concentration before reaching a plateau, as shown in Figure S9. The supply rate of **HT** is high and its concentration asymmetry (Δ*C*) is maintained by the disk continuously relocating toward the region of high surface tension (Stage I). Over time, the amount of **HT** on the disk is consumed, resulting in a lower driving force. At this point, the supply rate is too low to counteract the combined effects of **HT** removal by dissolution and relocation, thus the disk abruptly slows to a halt (Stage II_a_). In the oscillatory mode, the processes of supply and removal do not happen simultaneously. This may be due to interactions between **HT** and the hydrophobic disk surface which allow **HT** to accumulate around the base of the disk, generating a region of reduced surface tension (Stage II_b_). Spontaneous symmetry breaking causes the disk to rapidly accelerate outside this region to an area of high surface tension (Stage II_c_). The motion prevents sufficient accumulation of **HT** and the disk comes to a halt before repeating the cycle. The frequency of the oscillatory mode decreases with time as the time it takes **HT** to diffuse from deeper within the matrix increases.

**Figure 5:**
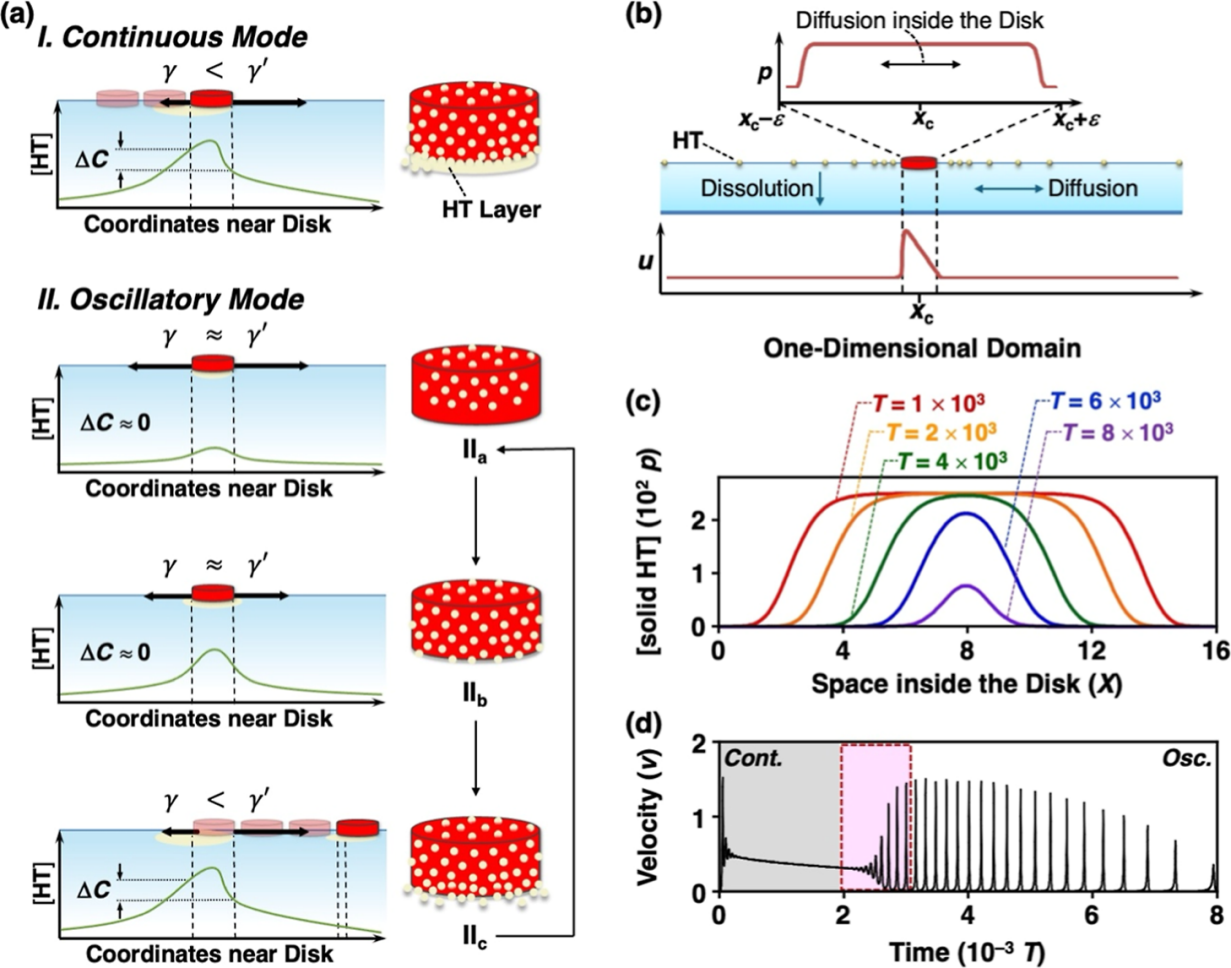
(a) Schematic illustration showing the plausible mechanism of sequential mode switching and the **HT** distribution in the **HT-SIS** disk. γ and γ′ represent the surface tension at the back and front sides of the disk, respectively. (b) Schematic illustration of the setup of the mathematical model, where *p*, *u*, *x*_c_, and ε represent the concentration of **HT** in the solid state, the surface concentration of **HT**, the position of the disk, and the radius of the disk, respectively. (c) Time evolution of the distribution of solid phase **HT** inside the disk. (d) Time series of the velocity of self-propelled motion. The gray, red, and white regions indicate continuous motion, transition, and oscillatory motion phases, respectively. Both results in (c) and (d) were obtained from numerical simulations.

In order to verify the above mechanism, we performed numerical calculations of the self-propelled motion of the **HT** disk. The model was constructed on the basis of a previous report^81^ on the fundamental mechanism behind the self-propelled motion of a solid disk sliding on a water surface, which is driven by the surface tension gradient, while incorporating both the dissolution of **HT** from the disk surface and the diffusion of **HT** from the polymer matrix to the surface (Figure 5b; see the Mathematical Modeling section in Supporting Information for details). In this model, the **HT** concentration within the disk changes over time (Figure 5c). Initially, given that the disk was filled with a sufficient amount of **HT**, continuous motion was observed. Over time, **HT** at the edges of the disk was consumed, leaving depleted regions. As a result, the surface **HT** concentration decreased, leading to a gradual reduction in the disk speed for *T* < 2 × 10^3^, after which the mode of motion transitioned to oscillatory mode via several small oscillations. During oscillatory motion, the disk initially loses its driving force due to the depleted **HT** near the edges. However, **HT** molecules are readily supplied from the disk interior and diffuse along the bottom surface of the disk. When these molecules reach the edges, they regenerated the driving force, resulting in a burst of rapid motion. The subsequent rapid movement again lowers the surface **HT** concentration beneath the disk, causing the disk to stop. This cycle of velocity oscillations was reproduced in our simulations as shown in Figure 5d. Eventually, nearly all **HT** is consumed, and the disk comes to a complete stop. The simulated velocity time series, which includes the transition from continuous motion to oscillatory motion, was in good agreement with our experimental results (Figure 2b–d and Supporting Information Movie 6).

To ascertain whether the mechanism of the motion mode switching is unique to **HT-SIS**, we conducted experiments by replacing **HT** with other self-propelling species. When tropolone—the parent compound of **HT**—was used, the **SIS** elastomer disk showed only a few seconds of continuous motion followed by one or two jumps before ceasing to move (Figure S10a and Supporting Information Movie 7). This result indicates the presence of the hydrophobic isopropyl moiety of **HT** is a crucial component for the sustained motion of **HT-SIS**. Substitution with camphor resulted in subtle rhythmic variations in speed, but none of the distinctive jumping motions occurred during the period of observation (Figure S10b and Supporting Information Movie 8). When camphoric acid was used, the **SIS** elastomer disk showed mode switching from continuous to oscillatory motion, however the behavior was less consistent and slower than **HT-SIS**. On average, the continuous mode lasted 11 ± 8 s and the average speed of the rhythmic jumping was 16 ± 15 mm s^–1^ with a frequency of 0.16 ± 0.14 s^–1^ in the first 300 s (Figure S10c and Supporting Information Movie 9). These results indicate there are properties of **HT** that make it particularly suited to inducing frequent, high-speed jumping motions after spontaneous transition from continuous motion mode when blended with the **SIS** elastomer scaffold.

We also measured the surface tension of these surfactants, or fuel species, because it is a crucial parameter that induces Marangoni flow.^79^ As mentioned above, the surface tension of aqueous solutions containing **HT** decreased before reaching a plateau, as shown in Figure S9a. By linearly approximating the change in surface tension in the low-concentration region, γ = γ_0_ – α­[**HT**], the α value for **HT** was obtained to be 4.8 N m^–1^ M^–1^. Similarly, the α values for tropolone, camphor, and camphoric acid were calculated as 0.07 N m^–1^ M^–1^, 5.0 N m^–1^ M^–1^, and 4.3 N m^–1^ M^–1^, respectively. With the exception of tropolone, the surface tension of these fuel species shows similar concentration dependence, implying that the concentration of **HT** in the aqueous surface layer must vary differently with time compared to the other species. To verify the role of ambient surface tension, we placed **HT-SIS** disks onto aqueous solutions containing sodium dodecyl sulfate (SDS), a typical surfactant previously used to induce oscillatory motion.^50^ Low concentrations were chosen in order to approximately mimic the local surface tension surrounding the disk at different **HT** concentrations (Figure S9b), though it should be noted that SDS has a greater propensity to remain on the surface compared to **HT**. At [SDS] of 0.5 mM the **HT-SIS** disk moved continuously for only a few seconds before jumping at irregular intervals (Figure S11a). At [SDS] of 1.0 mM and 2.0 mM, disks moved extremely slowly for more than 150 s before oscillating at frequencies of 0.04 s^–1^ and 0.03 s^–1^, respectively (Figure S11b,c). These frequencies are more than 10-fold smaller than that of disks in the absence of SDS, which suggests it takes longer for a sufficient driving force to emerge when the ambient surface tension is lowered. Remarkably, the behaviors exhibited in Figure S11 can all be considered spontaneous mode switching, which demonstrates the emergence of the oscillatory mode is not caused by reaching some ambient surface tension threshold, in contrast to an earlier report using camphoric acid in agarose gel.^58^ This implies the switch to rhythmic jumping behavior may be from temporal variations of [**HT**] as it is released.

To visualize how **HT** behaves on the surface as it leaves the disk, Quinzarin Green SS—a hydrophobic dye—was spread on the water surface before a **HT-SIS** disk was deposited. As **HT** emerges from the disk into the surface layer of water, it lowers the surface tension causing the dye particles to be pulled away. During the continuous mode, a small clear area was observed trailing the disk (Figure S12a). The size of this diffusion layer remained constant, indicating the supply rate of **HT** to the surface is roughly equivalent to its removal rate from the surface. Figure S12b depicts one cycle of the oscillatory mode. The supply of **HT** to the surface is paused during the rest state as indicated by the absence of an expanding diffusion layer surrounding the disk. In a fraction of a second, a cleared region forms on one side of the disk before it rapidly accelerates. As the disk begins to slow to rest again, the trailing diffusion layer shrinks or is left behind. The oscillation of local **HT** concentration—and therefore, surface tension—with respect to time may be explained by considering the balance between the amount of **HT** supplied from the **SIS** elastomer and the rate of surface diffusion and dissolution (removal).^56^

### Control of Motion Mode Using Different Polymer Matrices

The rate at which **HT** is supplied from within the polymer matrix to the surface—consequently affecting the oscillatory behavior—should be influenced by not only the phase states of **HT** (amorphous versus crystalline), but also the components of the polymer matrix.^82,83^ Therefore, we studied how the motion mode of **HT** disk is affected by the phase-separated structure of the polymer matrix. We systematically prepared **HT** disks with varying polystyrene content: a disk with 22 wt % polystyrene as described above (**HT-SIS**), a disk with 50 wt % polystyrene (**HT-SIS50**), and a polystyrene-only disk (**HT-PS**). **HT-SIS50** was prepared by blending high molecular weight polystyrene with the **SIS** elastomer to achieve 50 wt % styrene content (see Supporting Information for details).^84^ As the styrene content of the disks increased, the self-propelled behavior became less intense. **HT-SIS50** disks first displayed continuous motion for 45 ± 14 s on average, then oscillatory behavior between rest and sudden jumps that averaged 32 ± 5 mm s^–1^ with a frequency of 0.28 ± 0.09 s^–1^ during the first 300 s (Figure 2d, Table 1 and Supporting Information Movie 10). Meanwhile, **HT-PS** disks moved very slowly, showing no obvious continuous mode and irregular jumping motions that averaged 8 ± 9 mm s^–1^ during the first 300 s (Figure 2e, Table 1 and Supporting Information Movie 11).

Fourier transform infrared spectroscopy (FT-IR) exhibited shifts in the carbonyl and hydroxyl stretching frequencies when **HT** was mixed with various polymer matrices (Figure 6a), indicating interactions between **HT** and the polymer matrices analyzed. When blended with either **SIS** or polyisoprene (**PI**), the characteristic carbonyl stretching of **HT** shifted from 1606 cm^–1^ to 1614 cm^–1^. Peak splitting to 1612 cm^–1^ and 1602 cm^–1^ was observed when blended with polystyrene (**PS**). The hydroxyl stretching frequency shifted from 3184 cm^–1^ to 3198 cm^–1^ when blended with **PI** and 3194 cm^–1^ when blended with **SIS** or **PS**. These results indicate **HT** is distributed across both **PI** and **PS** domains. The substitution of tropolone blended with various polymers resulted in similar shifting and splitting of the carbonyl peaks, though no shift in frequency was observed for the hydroxyl stretching peak (Figure S13a). Camphor and camphoric acid also showed subtle shifting of their respective carbonyl peaks when blended with **SIS** (Figure S13b,c).

**Figure 6:**
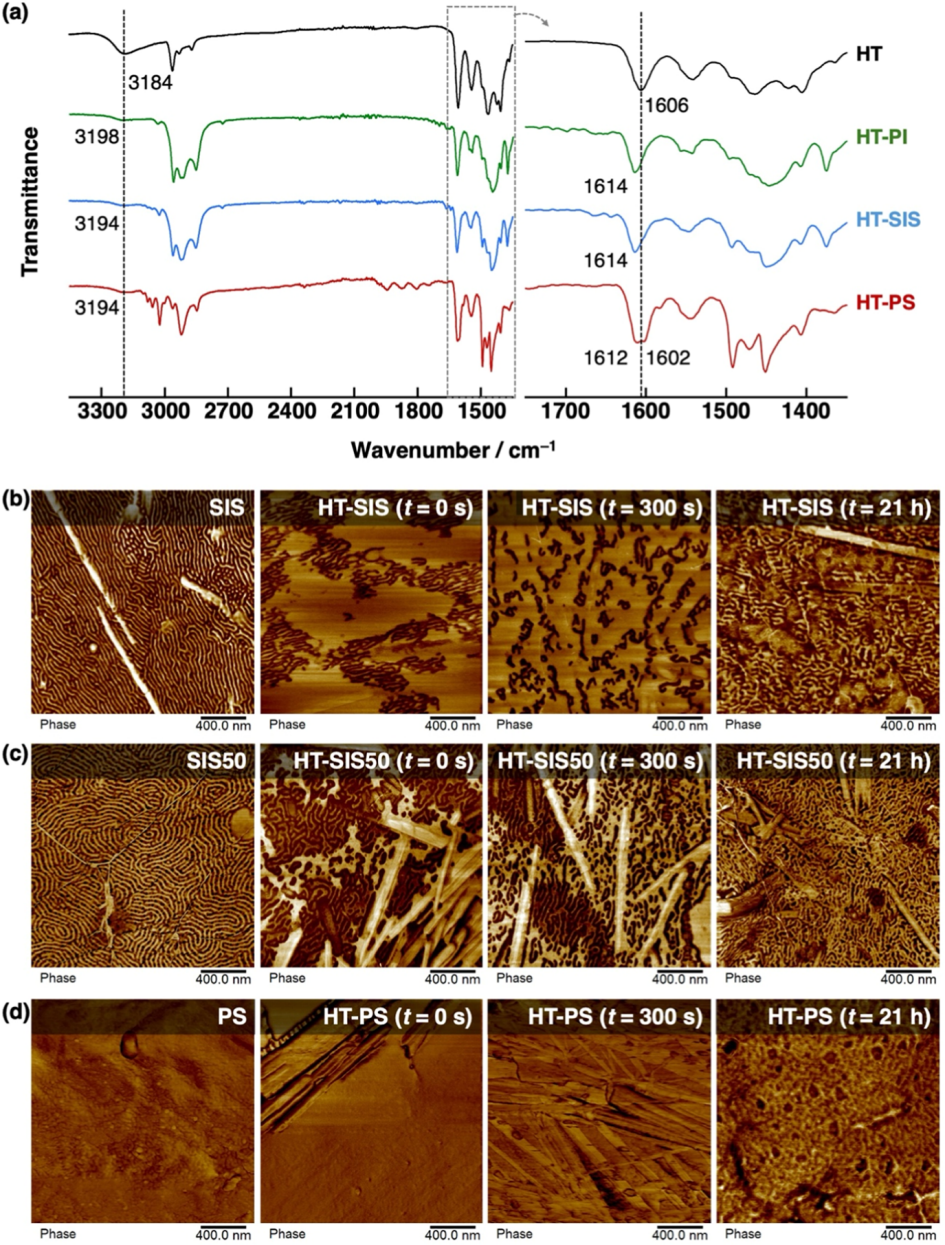
(a) FT-IR spectra of **HT** (black) and **HT** blended with either **PI** (green), **SIS** (blue), or **PS** (red). AFM phase images of (b) **HT-SIS**, (c) **HT-SIS50**, and (d) **HT-PS** disks before (center left) and after swimming for 300 s (center right) and 21 h (right) on distilled water at 25 °C. Each respective elastomer in the absence of **HT** is shown on the left. All samples contain 0.5 wt % visualizing dye.

The surface morphology of disks with varying styrene-isoprene ratios was observed using atomic force microscopy (AFM) operated in tapping mode. Phase images, rather than height images (Figure S14), were used to identify regions of differing stiffness and adhesion. The height and phase images of **HT-SIS** revealed large, relatively bright areas interspersed with worm-like dark domains (Figures 6b and S14a). After the disk was allowed to swim on distilled water for ca. 300 s the worm-like domains became more dispersed; after 21 h the worm-like domains became more densely packed and with average thicknesses similar to the bright regions of **SIS** in the absence of **HT**, albeit without its orderly lamellar structures. Notably, analogous disks constructed of other fuel species mixed with **SIS** did not show such drastic changes in morphology due to swelling of one domain when compared to **SIS** (Figure S14). This may be due to the ability of **HT** to remain in the liquid state after disk fabrication while the other fuel species do not. With the increased styrene ratio of the polymer matrix, the bright lamellae grew longer and thicker in **SIS50**; when blended as **HT-SIS50**, these lamellae were replaced by collections of highly complex morphologies (Figure 6c). Similar to the case of **HT-SIS**, the morphology of **HT-SIS50** after 21 h appeared as densely packed, bright worm-like structures instead of the orderly lamellae of the **SIS50** matrix. This suggests **HT** can affect the interfacial properties of **PI** and **PS** domains. No microphase separation was observed in **HT-PS**, which was in agreement with the FT-IR results suggesting miscibility between **HT** and polystyrene (Figure 6d).

Force curves were also measured to confirm the molecular composition and to characterize the mechanical properties of the bright and dark domains in each phase image (Figure S15).^85–87^ The bright regions recorded on a fresh **HT-SIS** disk showed a strong adhesive force and significant hysteresis compared to the dark worm-like regions (Figure S15a). After swimming for 300 s the adhesive force of the bright region appeared to weaken while the sample remained compliant, which may be attributed to the consumption of **HT**. Force curves measured on **HT-SIS50** and **HT-PS** also showed changes in their stiffness and adhesive properties consistent with the release of **HT** after swimming (Figure S15b,c). The decrease in adhesive force and plastic deformation reflects several mechanistic implications. Initially, the local concentration of **HT** is high at the disk–water interface compared to the ambient water, causing a substantial decrease in local surface tension. Over the course of swimming, the amount of **HT** at the interface decreases causing the local surface tension to increase, which was confirmed by changes in water contact angles on the **HT-SIS** surface at various time intervals compared to that of **SIS** (Figure S16). The average value for fresh **HT-SIS** at 98.9 ± 4.3° was larger than that of **SIS** at 82.0 ± 0.6° which shows how the presence of **HT** weakens the interfacial tension between the disk and water relative to the surface tension of the water. After swimming on distilled water for 60 s, 300 s, or 17 h, the contact angles progressively decreased to 97.6 ± 1.6°, 94.2 ± 1.3°, and 84.5 ± 0.7°, respectively. Consequently, the surface tension gradient, Δγ, surrounding the disk becomes smaller and the disk speed decreases.

In the oscillatory mode, the disk cycles between states of rest as **HT** builds up and rapid escape; therefore, the scaffold material can be used to modulate this buildup step based on how well it permits **HT** to diffuse from deeper within.^88^ Although **HT** was found to be miscible with both blocks, the greatest morphological and mechanical changes were observed in the bright domain of **HT-SIS**, when the isoprene content was highest, implying a preferential release pathway through domains constructed from the more flexible material. Conversely, the presence of interpenetrating **PS** domains may suppress the supply rate of **HT**. At 22 wt % styrene, the **HT**-swollen **PI** domain forms a large, continuous network that initially covers 79.8% of the field of view in Figure 6b, as analyzed using ImageJ (see Supporting Information for details). After swimming for 300 s, that surface coverage shrunk to 74.5% but maintained its connectivity, thus permitting multiple release pathways for rapid **HT** buildup during the oscillatory mode of **HT-SIS**. In the case of **HT-SIS50**, the surface area of the **PI** domain was reduced to ca. 50% of the field of view in Figure 6c. The patches where globular bright areas transition into less bright, worm-like structures imply that **HT** may not be consistently distributed in each polymer block, further limiting potential release pathways. This diminished surface coverage suggests the shorter continuous mode observed for **HT-SIS50** may be attributed to a smaller initial **HT** layer compared to **HT-SIS**. After swimming for 300 s, the surface coverage of the soft domains decreased to ca. 35% while becoming more dispersed. As a result of fewer release pathways available, **HT-SIS50** required longer **HT** buildup times resulting in lower-frequency oscillations. The amount of **HT** fuel accumulated in each cycle from **HT-SIS50** may also have been diminished, which would account for slower average jump speeds compared to **HT-SIS**. The molecular size of the polymer chains themselves may also play a decisive role. In the case of **HT-SIS**, the relatively short styrene blocks have fewer opportunities to entangle, which may allow **HT** to easily permeate both domains. By contrast, the large chain lengths used to construct rigid **HT-PS** disks can more effectively constrain **HT** within the scaffold. When both types are combined, as in the case of **HT-SIS50**, the surface became more rigid after swimming which may hinder the continued diffusion of **HT**. Thus, the release of fuel species may be engineered via scaffold matrix design.

## Conclusion

To conclude, we demonstrated here, for the first time, that **HT** exhibited self-propelled motion on water, and transition of motion modes—from continuous to oscillatory—when mixed with polystyrene elastomer matrices, even in the absence of any external input. By exploiting the unique property of **HT**, which can exist either as an amorphous oil or as a crystalline solid at room temperature depending on its thermal history, we were able to modulate both the duration of the continuous motion and the frequency of the oscillatory motion. Furthermore, blending the polystyrene elastomer with high molecular weight polystyrene enabled us to further regulate the diffusion rate of **HT** through the polymer matrix, which consequently affecting the oscillatory behavior. Our results underline that sophisticated self-propelled behaviors can be rationally designed by considering not only the molecular arrangement of the fuel species but also its interaction with the surrounding polymer scaffold. These insights present a novel strategy for the molecular-level design and control of macroscopic self-propelled behaviors, providing new perspectives for the development of synthetic life-like materials based on the interplay of fuel diffusion, phase transitions, and polymer microstructure.

## Supplementary Material

























## Notes

Detailed experimental methods, Supporting Information, and additional references (PDF)
Movie S1. Motion of a clump of **HT** crystals when floated on distilled water at 25 °C (MP4)
Movie S2. Motion of a **HT-SIS** disk when floated on distilled water at 25 °C (MP4)
Movie S3. Behavior of a **SIS** disk coated with **HT** when floated on distilled water at 25 °C (MP4)
Movie S4. Behavior of a **SIS** disk containing no active species when floated on distilled water at 25 °C (MP4)
Movie S5. Motion of a **cHT-SIS** disk when floated on distilled water at 25 °C (MP4)
Movie S6. Simulation of the time evolution showing (left) the surface concentration of **HT** (*u*) as a function of the disk position (*x*) and (right) the distribution of solid phase **HT** inside the disk (*p* vs *X*)­(MP4)
Movie S7. Motion of a tropolone-**SIS** disk when floated on distilled water at 25 °C (MP4)
Movie S8. Motion of a camphor-**SIS** disk when floated on distilled water at 25 °C (MP4)
Movie S9. Motion of a camphoric acid-**SIS** disk when floated on distilled water at 25 °C (MP4)
Movie S10. Motion of a **HT-SIS50** disk when floated on distilled water at 25 °C (MP4)
Movie S11. Motion of a **HT-PS** disk when floated on distilled water at 25 °C (MP4)
