# Supplementary material for: Autonomous Switching of Self-Propelled Motion Modes of Hinokitiol-Fueled Elastomer Matrices
Source: J Am Chem Soc. 2025 Oct 20;147(43):40024–34. doi: 10.1021/jacs.5c17346 (PMC12576836; doi:10.1021/jacs.5c17346)
Supplement: Supplementary file 1 [file ja5c17346_si_001.pdf]

Supporting Information

Autonomous Switching of Self-Propelled Motion Modes of Hinokitiol-Fueled Elastomer Matrices

Lara Rae Holstein,[1,2] Masayuki Takeuchi,[1,2] Nobuhiko J. Suematsu,*[3] and Atsuro Takai*[1,2]

[1] Molecular Design and Function Group, National Institute for Materials Science (NIMS), 1-2-1 Sengen, Tsukuba, Ibaraki 305-0047, Japan

[2] Department of Materials Science and Engineering, Faculty of Pure and Applied Sciences, University of Tsukuba, 1-1-1 Tennodai, Tsukuba, Ibaraki 305-8577, Japan

[3] School of Interdisciplinary Mathematical Sciences; Graduate School of Advanced Mathematical Sciences; Meiji Institute for Advanced Study of Mathematical Sciences (MIMS), Meiji University, 4-21-1, Nakano, Tokyo 164-8525, Japan

Table of Contents

Experimental Methods Page S2

Supporting Data

1. Sublimation Behavior of **HT** (**Figure S1**) Page S8
2. Friction Coefficient of **HT-SIS** Disks (**Figure S2**) Page S9
3. Self-Propelled Behavior of Fresh and Used **HT-SIS** Disks (**Figure S3**) Page S10
4. Self-Propelled Behavior of **SIS** Disks (**Figure S4**) Page S11
5. Thermal Properties of **HT** and **SIS** Elastomer (**Figure S5**) Page S12
6. XRD Pattern (**Figure S6**) Page S13
7. Dissolution Rate Analysis (**Figure S7** and **Table S1**) Page S14
8. Self-Propelled Behavior in Cold Water (**Figure S8**) Page S16
9. Self-Propelled Behavior of Other Fuel Species (**Figure S9**) Page S17
10. Surface Tension (**Figure S10**) Page S18
11. Self-Propelled Behavior in the Presence of SDS (**Figure S11**) Page S19
12. Diffusion Layer Analysis (**Figure S12**) Page S20
13. FT-IR Spectra (**Figure S13**) Page S21
14. AFM Height Images (**Figure S14**) Page S22
15. AFM Phase Images Using Other Fuel Species (**Figure S15**) Page S23
16. AFM Force Curves (**Figure S16**) Page S24
17. Water Contact Angles (**Figure S17**) Page S26

Author Contributions Page S27

Supporting References Page S27

Experimental Methods

Materials: Unless otherwise noted, all reagents and solvents were purchased from Tokyo Chemical Industry, Sigma-Aldrich, Wako Pure Chemical Industries, and Kanto Chemical and used without further purification. Distilled water was obtained from Kyoei Pharmaceutical. Polystyrene-block-polyisoprene-block-polystyrene (**SIS**, 22 wt % styrene) and high molecular weight polystyrene (**PS**, average M_w 350 kDa) were purchased from Sigma-Aldrich. *N*,*N*'-Bis(3-pentyl)perylenediimide (**PDI**) dye was synthesized according to the literature.[S1]

Preparation of Disks with Polymer Matrix: A chloroform ($CHCl_3$) solution of **SIS** (40 mg/2 mL) was mixed with a $CHCl_3$ solution of surface-active species (hinokitiol: **HT**, tropolone, camphor, or camphoric acid) and **PDI** as a visualizing dye in a 4:1:0.025 weight ratio. The same procedure was used to prepare **HT-SIS50** and **HT-PS** disks by substituting **SIS50** and **PS**, respectively. **SIS50** was prepared by blending **SIS** with **PS** such that the styrene content was 50 wt %.[S2] The mixed solution was spread on a Teflon Petri dish to evaporate $CHCl_3$, and placed into a square-shaped mold (approximately 10 × 10 mm^2, 0.5 mm thickness) cut in a silicone sheet resting on a Teflon plate. The silicone sheet was sandwiched with another Teflon plate, wrapped in aluminum foil, and pressed (0.9 kN) at 52 °C for 10 min, which completely removed the solvent and yielded the amorphous **HT-SIS** sheet. To obtain the **cHT-SIS** form, the amorphous sheet, still in its mold and wrapping, was placed in a watch glass, sealed to keep it dry, and placed in a cold methanol–liquid nitrogen bath adjusted to approximately –60 °C for 10 min. Elastomer sheets were stored on the bench top wrapped in foil and used one day after preparation. Uniform disks (2 mm diameter) were obtained by using a circular hole punch immediately before use. The mass of the disk was 1.4 ± 0.04 mg, and the amount of surface-active species per disk was *ca.* 0.2 mg.

Videography and Video Analysis: The movement of disks was monitored from above using a video camera (EOS Kiss X10, Canon; 30 fps), which was fixed to a copy stand (CS-A4, LPL). Distilled water (10 mL, 3 mm in depth) was added to a Petri dish (75 mm in diameter) and placed on a flat light panel (white LED light; A4-500, Trytec) to enhance the contrast. These setups were placed in a box partitioned with a black vinyl sheet to block air convection from the air conditioner, maintaining the room temperature at 25 ± 3 °C and humidity at 23 ± 8%. Although it has been reported that self-propelled motion can be affected by temperature and humidity,[S3] the variation under these conditions is reflected in the error bars presented in Table 1. The self-driven behavior

of the disk was evaluated under these conditions. The observation of self-propelled motion was conducted at least 6 times using fresh materials, including disks prepared from different batches. Due to melting during the fabrication process, there was a tendency for more **HT** to be present on the bottom side of the elastomer sheet, resulting in the top and bottom of each disk showing different continuous mode duration times depending on which surface was in contact with the water. To ensure consistent experimental conditions, we used only data obtained when the bottom side of the disks were in contact with the water for analysis. Parameters such as speed and frequency were statistically calculated. The obtained movie data were analyzed using ImageJ (NIH, USA).

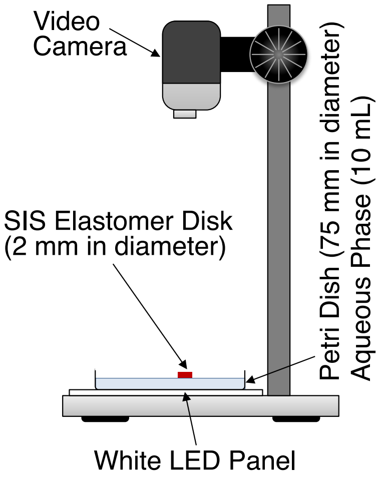

Polarized Optical Microscopy (POM) Measurement: The melting of fuel species and surfaces of polystyrene elastomer disks were observed by Olympus BX51, equipped with a digital camera (Olympus DP73) and a heating stage (Mettler Toledo HS82).

Thermal Analyses: Differential scanning calorimetry (DSC) measurement was carried out on a TA Instruments DSC250 under nitrogen atmosphere. The sample was encapsulated in a sealed aluminum pan. The DSC data were obtained during the second heating/cooling cycles at a scan rate of 10 °C min^{-1}. The sublimation behavior was investigated by thermogravimetric (TG) analysis using a NETZSCH STA2500 Regulus. Samples were placed in an aluminum pan and the time-dependent changes in sample weight were monitored at 45 °C under a flow of nitrogen gas.

X-ray Diffraction (XRD) Measurement: XRD patterns were recorded on a Rigaku MiniFlex 300/600 diffractometer equipped with D/teX Ultra X-ray detector. The Cu Kα X-ray radiation (λ = 1.5418 Å) was generated by Cu-rotator anode with operating voltage and current as 40 kV and 15 mA, respectively. The samples were prepared as elastomer sheets following the disk fabrication procedure. Data were collected in the range of 2θ = 2° to 40° with a scan speed of 4° min^{-1} at 25 °C.

Surface Tension Measurement: The surface tension at solid–water interface was measured by the Wilhelmy method using a DyneMaster DY-300 (Kyowa Interface Science Co., Ltd.). A sample solution (20 mL) was poured into a 50 mL Petri dish (60 mm in diameter), and a Pt plate (23.85

mm in width and 0.15 mm in thickness) was used as the detector. The measurements were performed at least five times, and the average values were used.

Fourier Transform Infrared (FT-IR) Spectroscopy: FT-IR spectra were acquired on a JASCO FT/IR 4700 spectrophotometer equipped with an attenuated total reflection (ATR) attachment. The pristine fuel species were measured fresh from the bottle and fuel-polymer blend samples were prepared the same way as disks. The spectrum of each sample was recorded by 64 scans.

Atomic Force Microscopy (AFM): The surface morphologies of polymer samples were obtained by a Bruker Dimension Icon equipped with either a ScanAsyst-Air probe (k = 0.4 N m^{-1}) for softer elastomer samples, or a OTESPA-R3 probe (k = 26 N m^{-1}) for hard **PS** samples, operated in tapping mode using moderate to light tapping. Force curves were measured in ScanAsyst mode using a clean ScanAsyst probe. The deflection sensitivity was calibrated using the sapphire-12M sample supplied by Bruker. AFM samples were prepared by fixing disks to a steel stage with adhesive tape. At least three points were measured for each sample to ensure reproducibility. The area ratio between the bright and dark regions in the AFM images was precisely analyzed using the "Analyze Particles" function in ImageJ (NIH, USA).

Water Contact Angle Measurement: The static drop method was employed using a VCA Optima contact angle system. All measurements were performed at five different locations on each sample.

Dissolution Rate Analysis: When a **HT** disk with a 2 mm diameter was placed on 3 mL of distilled water in a 1.0 cm quartz cuvette, the **HT** dissolved into the bulk water over time, which was monitored by UV-vis absorption spectra. UV-vis absorption spectra were recorded on a JASCO V-630 spectrophotometer using time-interval measurement mode while stirring the sample solution at 600 rpm.

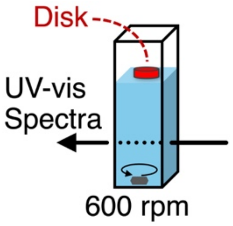

The dissolution rate of **HT** at the aqueous–disk interface can be described using the typical empirical Noyes-Whitney equation.[S4,5] Given that the absorbance at a certain wavelength (A_x) is only derived from the absorption due to **HT**, the change in A_x over time could be fitted with the Noyes-Whitney equation combined with the Lambert-Beer's law, as shown in Equation S1:

$$A_x = A_s(1 - e^{-kt}) \quad \text{(S1)}$$

where A_s is the absorbance when the amount of dissolved **HT** reaches saturation and k is the rate constant. As suggested in the mechanism of motion mode switching, the dissolution rate constant may change over time due to the switching of motion mode. After a long time, the dissolution rate may approach nearly constant values that can be estimated using Equation S1. On the other hand, the initial dissolution rate constant of **HT** (k_{ini}) was determined by linear approximation of the initial portion of the dissolution curve, assuming that the bulk concentration was negligible. Specifically, the expression $(1 - e^{-kt})$ in Equation S1 can be approximated as (kt), which is represented as Equation S2.

$$A_x = A_s\, k_{ini}\, t \tag{S2}$$

Therefore, k_{ini} was obtained from the slope of the linear fit divided by the value of A_s, which was determined by fitting the data with the Noyes-Whitney equation.

Mathematical Modeling: The simplest mathematical model for the self-propelled motion of a solid disk sliding on a water surface, which is driven by the surface tension gradient, is based on the following differential equations (Equations S3 and S4).[S6]

$$m\frac{dv}{dt} = -\mu v - \alpha\big(u(x_c + \varepsilon) - u(x_c - \varepsilon)\big)\varepsilon \tag{S3}$$

$$\frac{\partial u}{\partial t} = D\frac{\partial^2 u}{\partial x^2} - ku + S(x) \tag{S4}$$

The velocity of the disk (v) can be described by the Newton equation as shown in Equation S3, where m, μ, α, x_c, and ε denote the mass of the disk [kg], the friction coefficient [kg s^{-1}], the surface activity [kg m^2 s^{-2} mol^{-1}], the position of the disk [m], and the radius of the disk [m], respectively. In this context, the first term on the right-hand side corresponds to the friction force, while the second term represents the driving force originating from surface tension $\gamma(x)$ [kg s^{-2}]. Given the linear relation $\gamma = \gamma_0 - \alpha[\mathbf{HT}]$, the driving force can be expressed as the difference in surface tension acting on both edges of the disk, which depends on the surface concentration of **HT** (u) [mol m^{-2}] as defined in Equation S4.

Equation S4 represents the reaction-diffusion equation for the surfactant on water: The first term describes the diffusion process, the second term represents dissolution, and the third term

accounts for the supply from the disk, where D is the diffusion coefficient [m^2 s^{-1}] and k is the dissolution rate constant [s^{-1}]. The supply rate, $S(x)$, depends on the space. In our experiment, $S(x)$ may also vary with time, because the self-propelled disk comprises the polymer matrices as the supporting medium for **HT**, and the **HT** concentration within the disk changes over time. Therefore, we modified the previous model by introducing a calculation for the time evolution of the internal solid phase of **HT** using the following partial differential equations (Equations S5 and S6);

$$\frac{\partial w}{\partial t} = D_w \frac{\partial^2 w}{\partial x^2} - k_{ad} wp + k_{dis} p \tag{S5}$$

$$\frac{\partial p}{\partial t} = k_{ad} wp - k_{dis} p \tag{S6}$$

where D_w, w, and p represent the diffusion coefficient of the **HT** aqueous solution within the polymer matrix [m^2 s^{-1}], the concentrations of **HT** in the aqueous solution and solid state [mol L^{-1}], respectively. The solid **HT** dissolves at a rate $k_{dis}p$, producing aqueous **HT**. A fraction of **HT** in aqueous solution can re-adsorb to the solid with a rate $k_{ad}wp$. The solid phase of **HT** is assumed to have no diffusion, whereas only the aqueous solution diffuses. The Dirichlet boundary conditions with value zero are used for w and p. Consequently, the amount of **HT** inside the disk decreases over time due to diffusion. The supply function, $S(x)$, in Equation S4 is expressed with the supply rate of solid-state **HT**, as shown in Equation S7;

$$S(x) = \begin{cases} \beta p(X) & (x_c - \varepsilon \le x \le x_c + \varepsilon) \\ 0 & (x < x_c - \varepsilon, or x_c + \varepsilon < x) \end{cases} \tag{S7}$$

where β is a positive constant [10^{-3} m s^{-1}] and X is the space inside the disks that is defined as $X = x - (p_x - \varepsilon)$. Here, we assume that the supply rate is proportional to the concentration of solid phase **HT**. This implies that the concentration of **HT** in the aqueous solution at the contact with the disk is lower than the saturation concentration. Thus, the adsorption of **HT** molecules from solution to the solid is negligible.

The Equations S3–S7 can be rewritten using dimensionless parameters and variables as follows:

$$\rho \frac{dV}{d\bar{t}} = -V - \left(U(\bar{x}_c + \epsilon) - U(\bar{x}_c - \epsilon)\right) \quad \text{(S8)}$$

$$\frac{\partial U}{\partial \bar{t}} = \frac{\partial^2 U}{\partial \bar{x}^2} - u + \bar{S}(\bar{x}) \quad \text{(S9)}$$

$$\frac{\partial W}{\partial \bar{t}} = \delta \frac{\partial^2 W}{\partial \bar{x}^2} - \kappa_{ad} WP + \kappa_{dis} P \quad \text{(S10)}$$

$$\frac{\partial P}{\partial \bar{t}} = \kappa_{ad} WP - \kappa_{dis} P \quad \text{(S11)}$$

$$\bar{S}(\bar{x}) = \begin{cases} P(\bar{X}) & (\bar{x}_c - \epsilon \leq \bar{x} \leq \bar{x}_c + \epsilon) \\ 0 & (\bar{x} < \bar{x}_c - \epsilon, or \bar{x}_c + \epsilon < \bar{x}) \end{cases} \quad \text{(S12)}$$

The Dirichlet boundary conditions with value zero are adopted to Equations S10 and S11, and the periodic boundary conditions are to Equations S8 and S9. The initial conditions are set as $U = 0$, $V = 0.001$, and $\bar{x}_c = \Lambda/2$, $W = 0$, and $P = 5.0 \times 10^{-1}$. The numerical results shown in Figures 5c and 5d were obtained using the following parameters: $\rho = 5$, $\delta = 5.0 \times 10^{-3}$, $\kappa_{ad} = 0.56$, $\kappa_{dis} = 1.4 \times 10^{-2}$, $\epsilon = 8$, and $\Lambda = 200$. The dimensionless parameters are defined as follows:

$$\rho = \frac{\beta m}{\mu}, \delta = \frac{D_w k}{D\beta}, \kappa_{ad} = \frac{\mu k_{ad}}{\varepsilon \alpha \beta}\sqrt{kD}, \kappa_{dis} = \frac{k_{dis}}{\beta}$$

Supporting Data

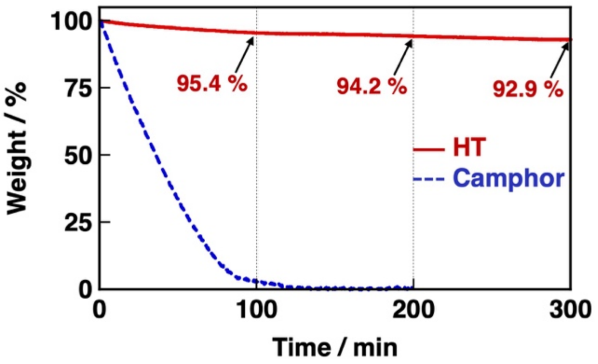

Figure S1. Time-dependent changes in the sample weights of **HT** and camphor at 45 °C under N_2 gas flow. The numbers at 100, 200, and 300 min indicate the remaining weight (%) at each time point. Compared to camphor, the sublimation rate of **HT** is significantly slower.

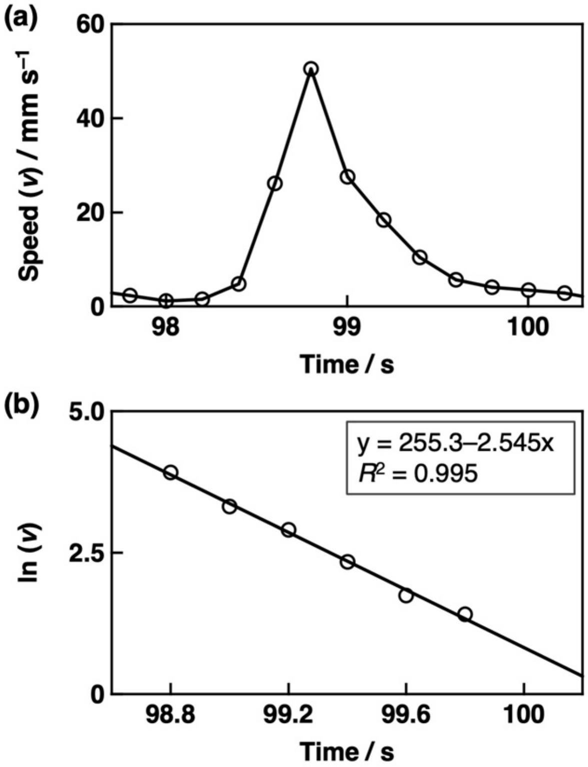

Figure S2. (a) Speed profile of a **HT-SIS** disk during one jumping cycle in the oscillatory mode. (b) Single logarithmic plot of speed (ln *v*) during the relaxation process (t = 98.8–99.8 s). The speed during the relaxation process of a single jumping event can be expressed as $v = v_0 \exp(-\mu t/m)$, where v_0, μ, m are the initial speed of the disk, the frictional coefficient, and the mass of the disk (1.4 ± 0.04 mg), respectively. The slope of ln (v) versus time corresponds to $-\mu/m$, and thus, μ can be determined by linear fitting. Similar plots were generated for the first five jumping events, and the average value of μ was obtained to be 3.46 (± 0.13) × 10^{-6} N m^{-1} s.

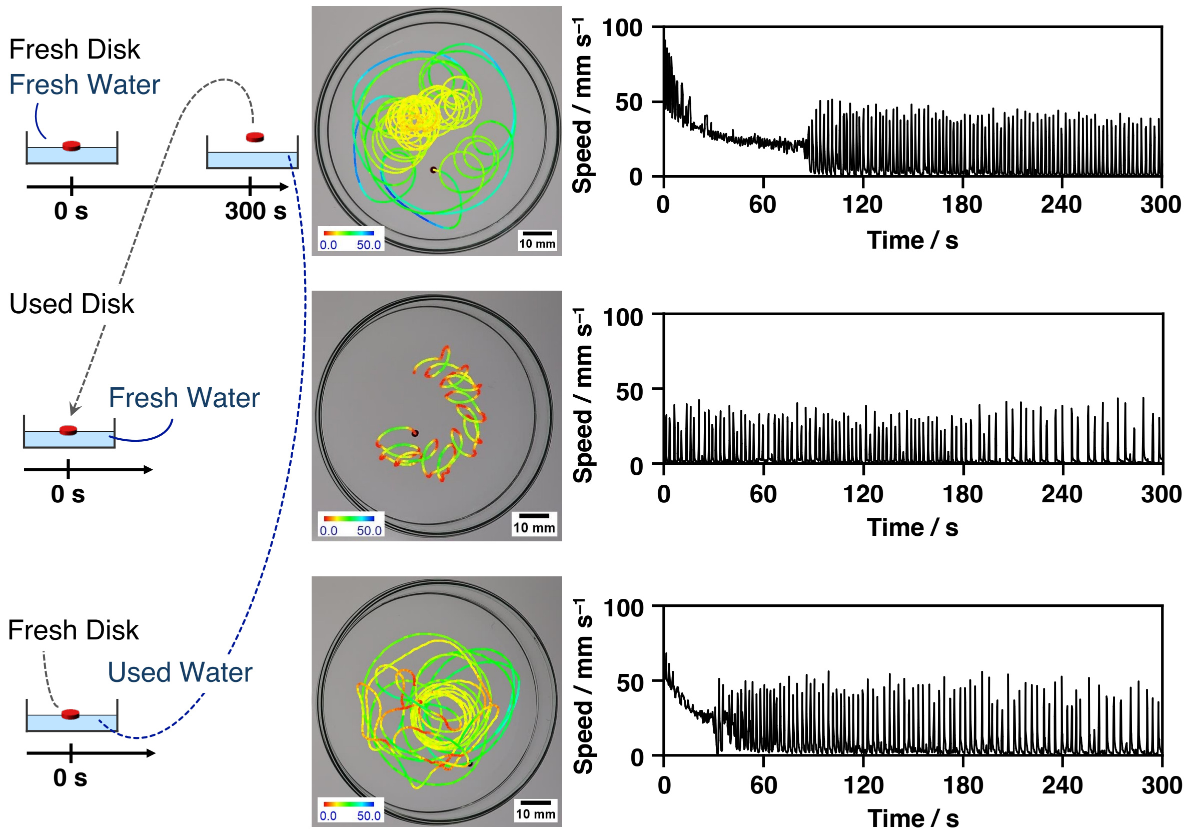

Figure S3. Schematic illustration (left) and the corresponding trajectory and speed profiles (right) of a **HT-SIS** disk allowed to swim for 300 s (top) before being transferred to fresh water (middle) and of a fresh disk placed on the used water (bottom). For clarity, the trajectory of only the first minute of motion was mapped.

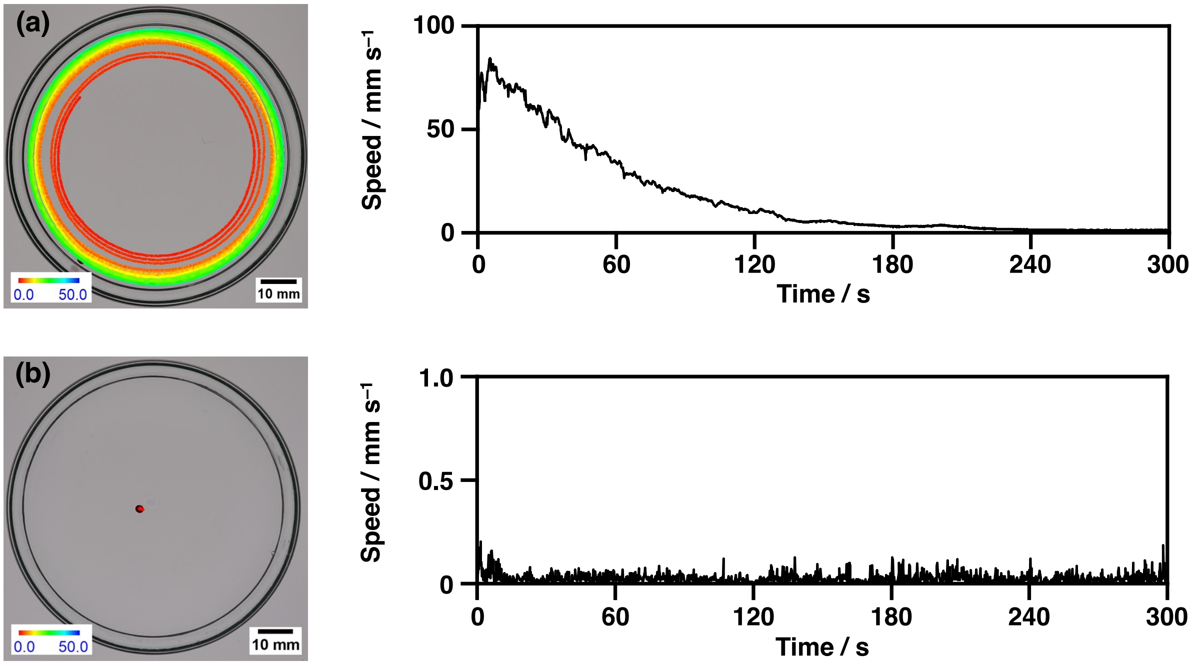

Figure S4. Trajectory and speed profile of (a) a **SIS** disk with **HT** coated on its surface and (b) a SIS disk in the absence of **HT**, each on distilled water at 25 °C.

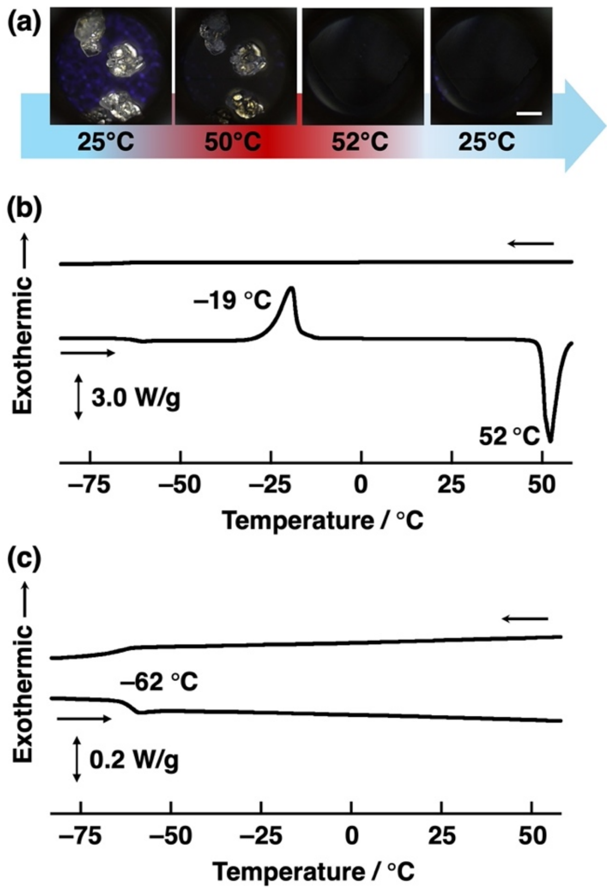

Figure S5. (a) POM images monitoring melting and cooling process of **HT**. Scale bar shows 500 µm. DSC profiles of (b) **HT** and (c) **SIS** elastomer during the second heating/cooling cycle at 10 °C min^{-1}.

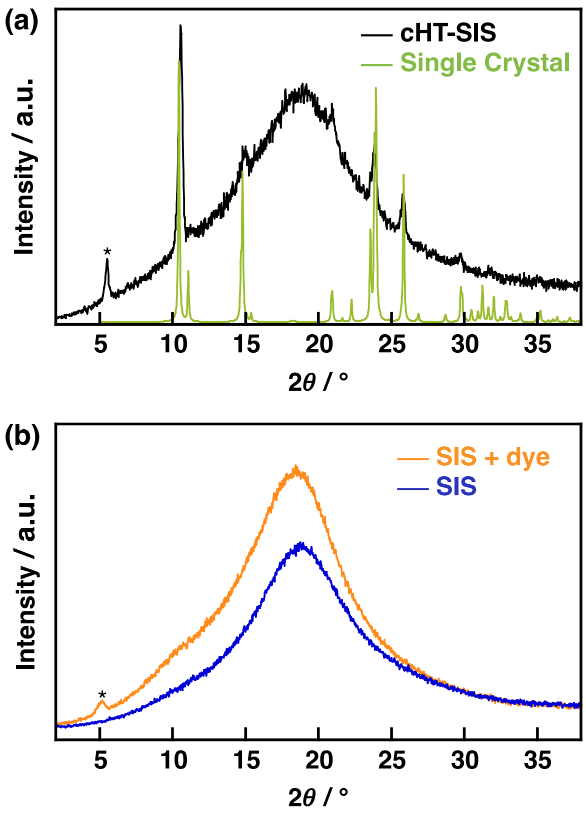

Figure S6. (a) XRD pattern of **cHT-SIS** (black) compared with the **HT** single crystal simulation (green).[S7] The crystallographic data (CCDC 1180774) was obtained from The Cambridge Crystallographic Data Centre *via* www.ccdc.cam.ac.uk/structures. (b) XRD patterns of the **SIS** elastomer with (orange) and without (blue) **PDI** visualizing dye. The asterisk at $2\theta = 5.6°$ (1.60 nm) denotes the peak corresponding to **PDI** assemblies.

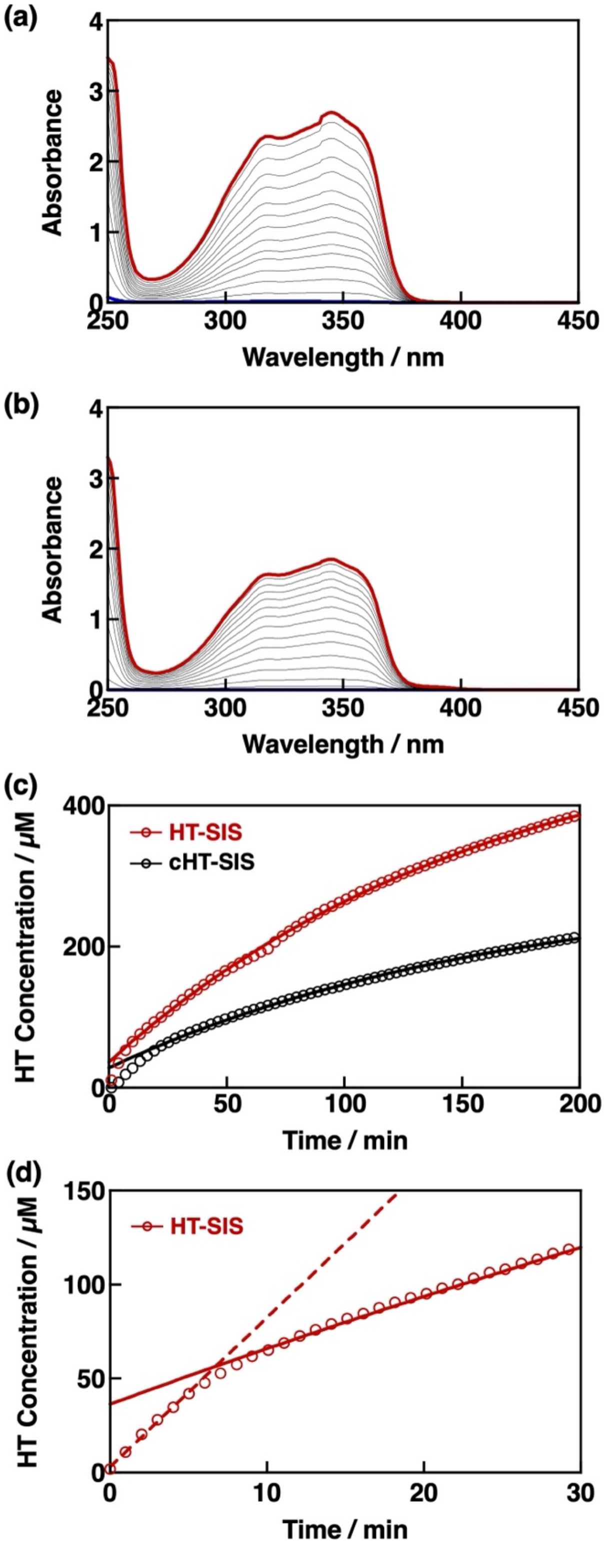

Figure S7. UV-vis absorption spectral changes observed upon addition of (a) **HT-SIS**, or (b) **cHT-SIS** disks to water at 25 °C. (c) Change in the **HT** concentrations calculated from UV-vis absorption spectral changes and the absorption coefficient at 345 nm (6.96×10^3 M^{-1} cm^{-1}). (d)

Enlarged area showing the initial change in **HT** concentration released from the **HT-SIS** disk. In (c) and (d), each solid line denotes the fitting curve obtained by the Noyes-Whitney equation with an R^2 value over 0.99 (see Page S4–S5; Dissolution Rate Analysis for details). The experimental values in the first 6 min noticeably increased more rapidly than the kinetics predicted from the fitting curve, indicating that the dissolution rate of **HT** from the disk decreased after an initial period. Indeed, a linear approximation (red dashed line) of the dissolution rate constant for the first 6 min yielded $k_{ini} = 2.68 \times 10^{-4}$ s^{-1}, which is clearly larger than the rate constant obtained by Noyes-Whitney fitting using the data collected after 8 min (k = 1.02 × 10^{-4} s^{-1}). This estimation is reasonable, considering the presence of an initial layer of **HT** on the disk surface responsible for the continuous motion. The dissolution rate constants and the saturated concentration (solubility) of **HT** are summarized in Table S1. The dissolution rate constants of **HT-SIS** were slightly higher, and its solubility was clearly greater than that of **cHT-SIS**.

Table S1. Dissolution Rate Constant and Solubility of HT Released from Disks into Water at 25 °C

disk name	dissolution rate constant / s^{-1}	solubility / μM
**HT-SIS**	k_{ini}: 2.68 (± 0.10) × 10^{-4}<br>k: 1.02 (± 0.01) × 10^{-4}	495.0 ± 2.7
**cHT-SIS**	k_{ini}: 2.10 (± 0.03) × 10^{-4}<br>k: 9.99 (± 0.17) × 10^{-5}	262.8 ± 2.1

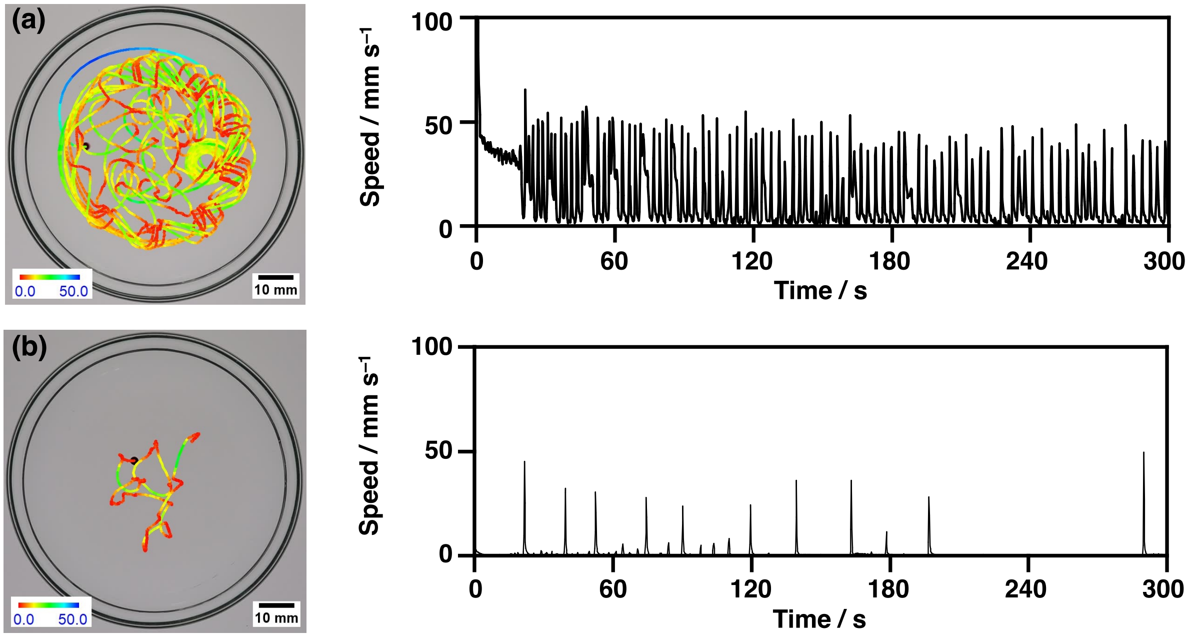

Figure S8. Trajectory and speed profiles of (a) **HT-SIS** and (b) **cHT-SIS** disks floated on distilled water at 5 °C. Compared to their behavior at room temperature, **HT-SIS** disks placed on cold water at 5 °C showed a shorter continuous mode duration of 18 ± 9 s and less frequent oscillations 0.3 ± 0.1 s^{-1} with an average jump speed of 34 ± 3 mm s^{-1}. By contrast, the behavior of **cHT-SIS** disks on cold water was drastically different from room temperature: displaying no continuous mode and significantly reduced frequency oscillations of 0.07 ± 0.01 s^{-1} with an average jump speed of 27 ± 9 mm s^{-1}.

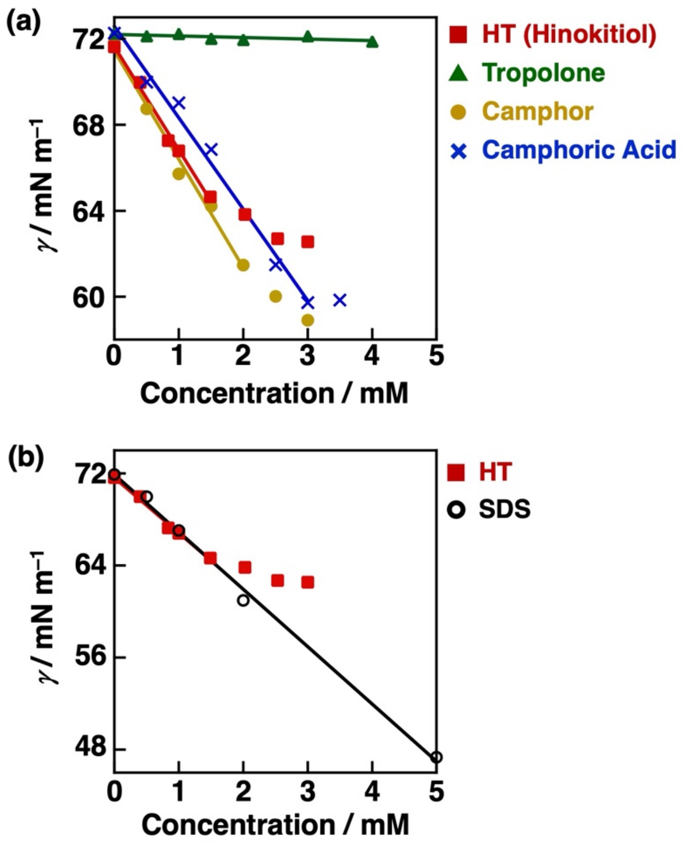

Figure S9. Concentration dependant surface tension (γ) measurements of (a) different fuel species (**HT**, tropolone, camphor, and camphoric acid) and (b) **HT** versus sodium dodecyl sulfate (SDS) in distilled water at 25 °C.

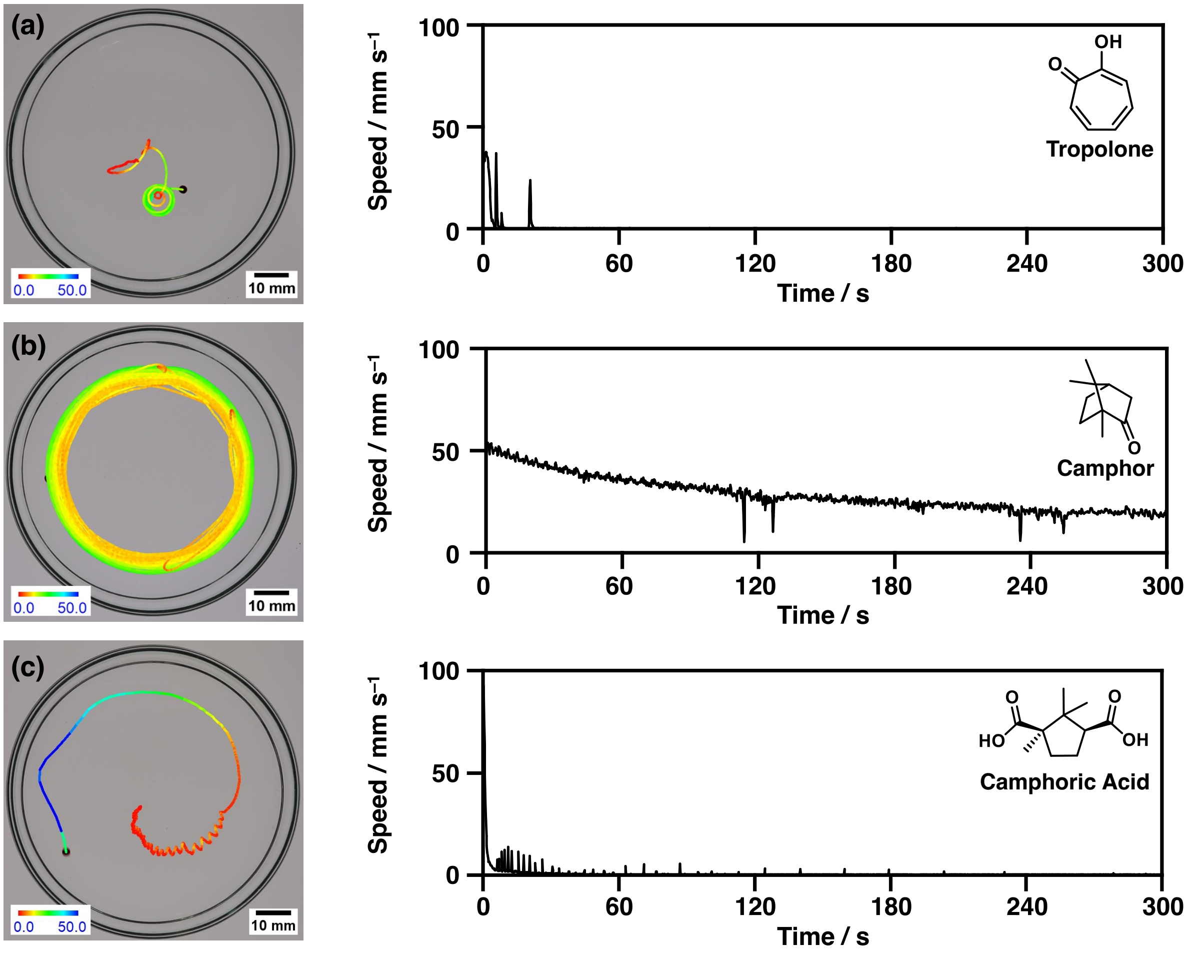

Figure S10. Trajectory and speed profiles of **SIS** elastomer disks containing either (a) tropolone, (b) camphor, or (c) camphoric acid.

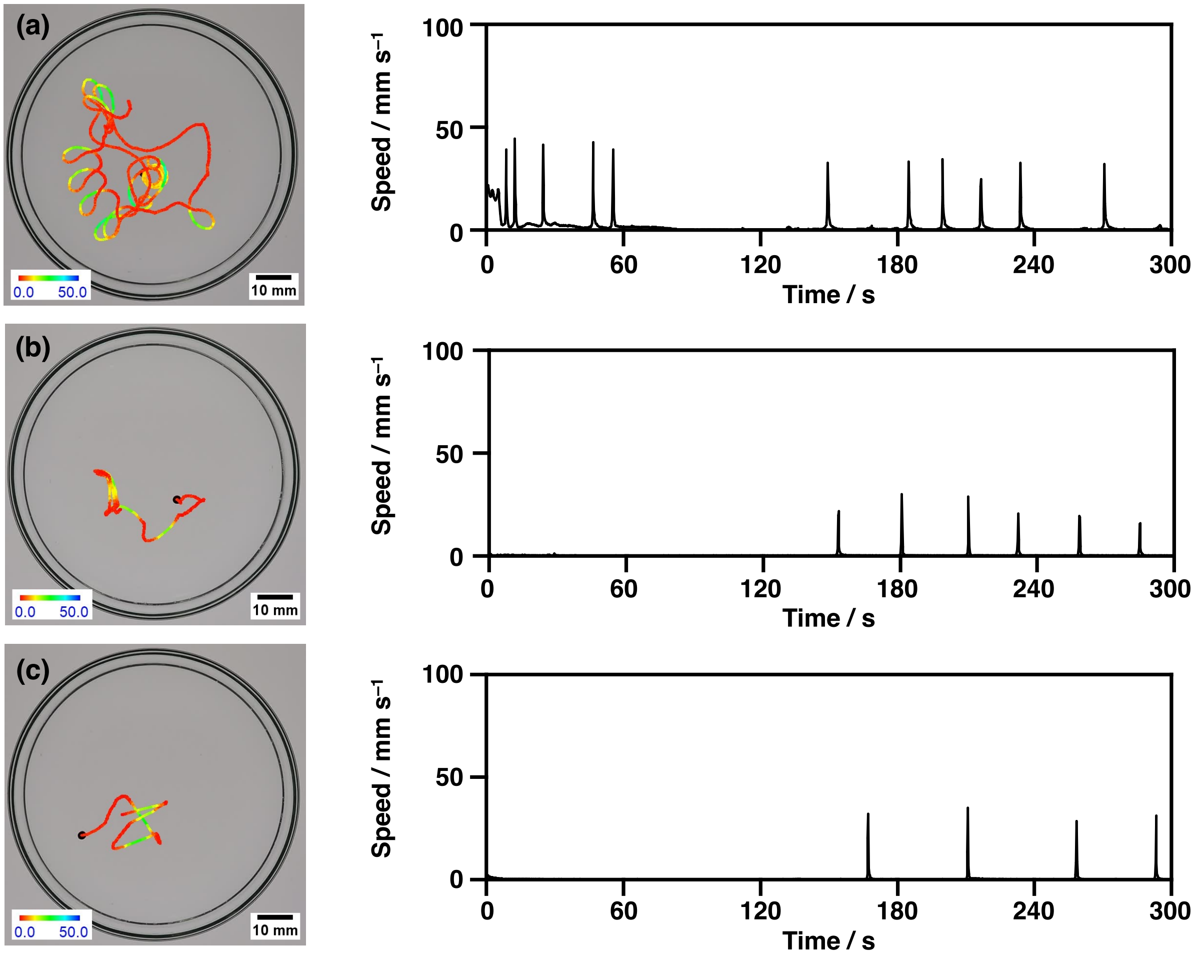

Figure S11. Trajectory and speed profiles of **HT-SIS** disks on aqueous solutions containing SDS at concentrations of (a) 0.5 mM, (b) 1.0 mM, and (c) 2.0 mM.

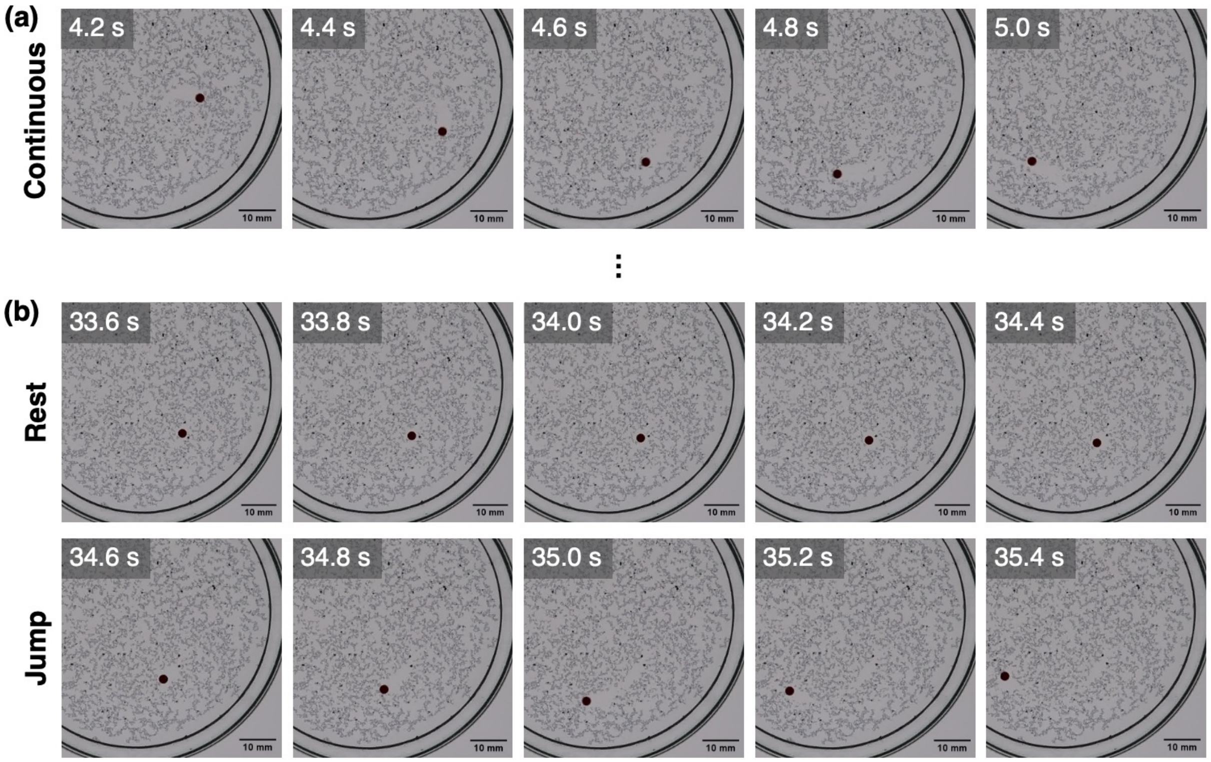

Figure S12. Snapshots of the development of a **HT** diffusion layer as a **HT-SIS** disk first moves (a) continuously and then (b) oscillates between rest (top) and jumping (bottom). The water surface was coated with Quinzarin Green SS particles to visualize the diffusion dynamics. All scale bars represent 10 mm.

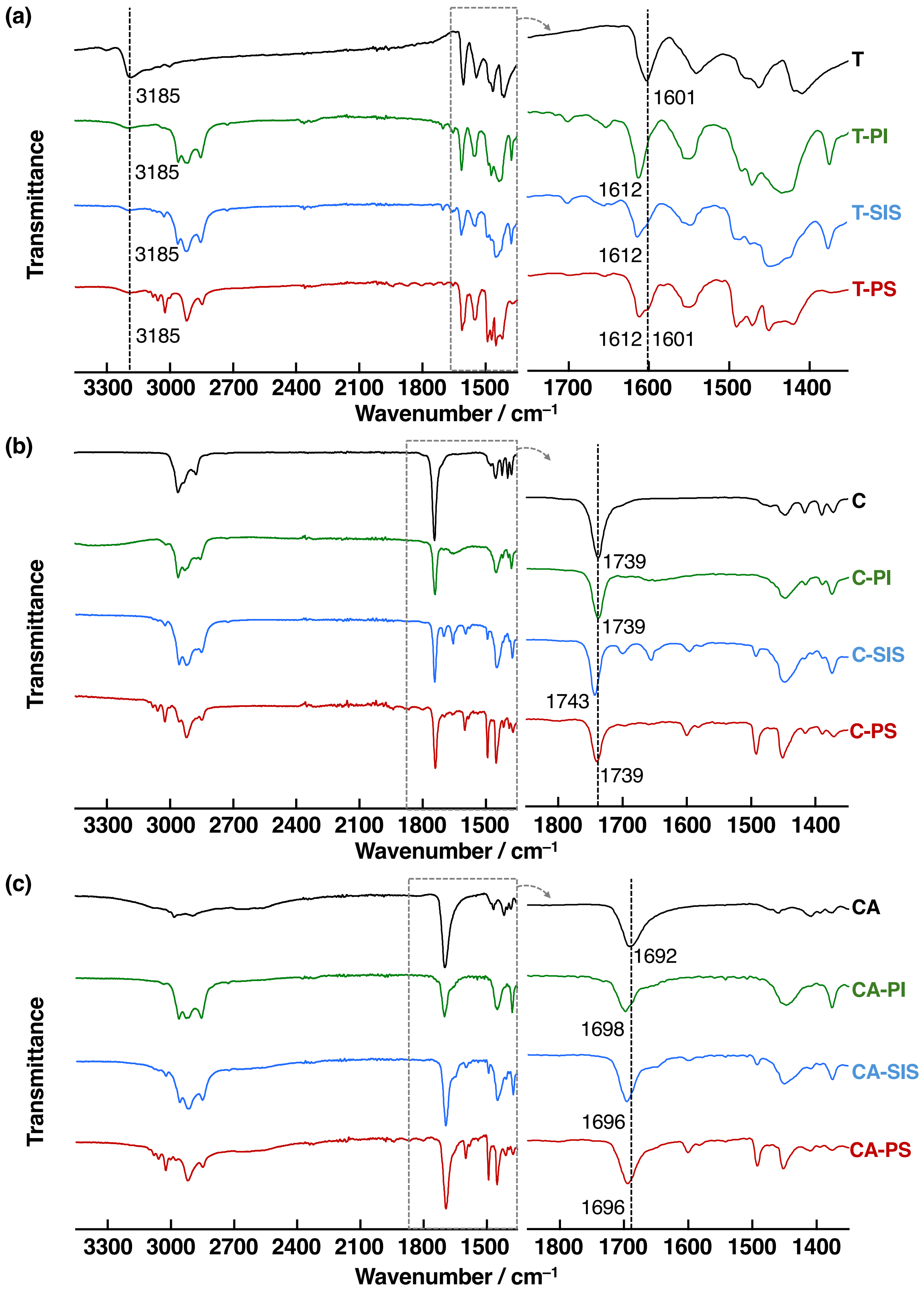

Figure S13. FT-IR spectra of (a) tropolone, (b) camphor, and (c) camphoric acid blended with either **PI** (green), **SIS** (blue), or **PS** (red). All samples contain 0.5 wt % **PDI** dye.

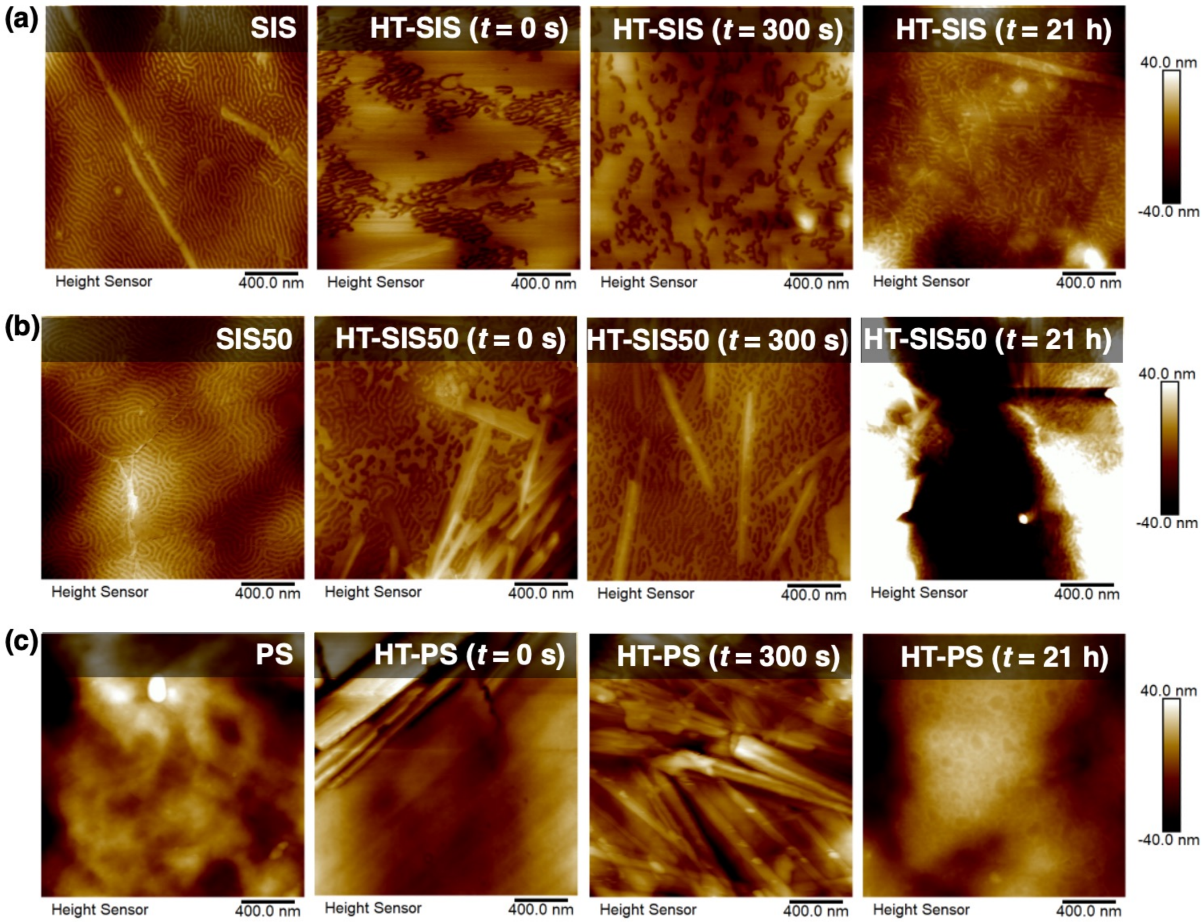

Figure S14. AFM height images of (a) **HT-SIS**, (b) **HT-SIS50**, and (c) **HT-PS** disks before (center left) and after swimming for 300 s (center right) and 21 hours (right) on distilled water at 25 °C. Each respective elastomer in the absence of **HT** is shown on the left. All samples contain 0.5 wt % **PDI** dye.

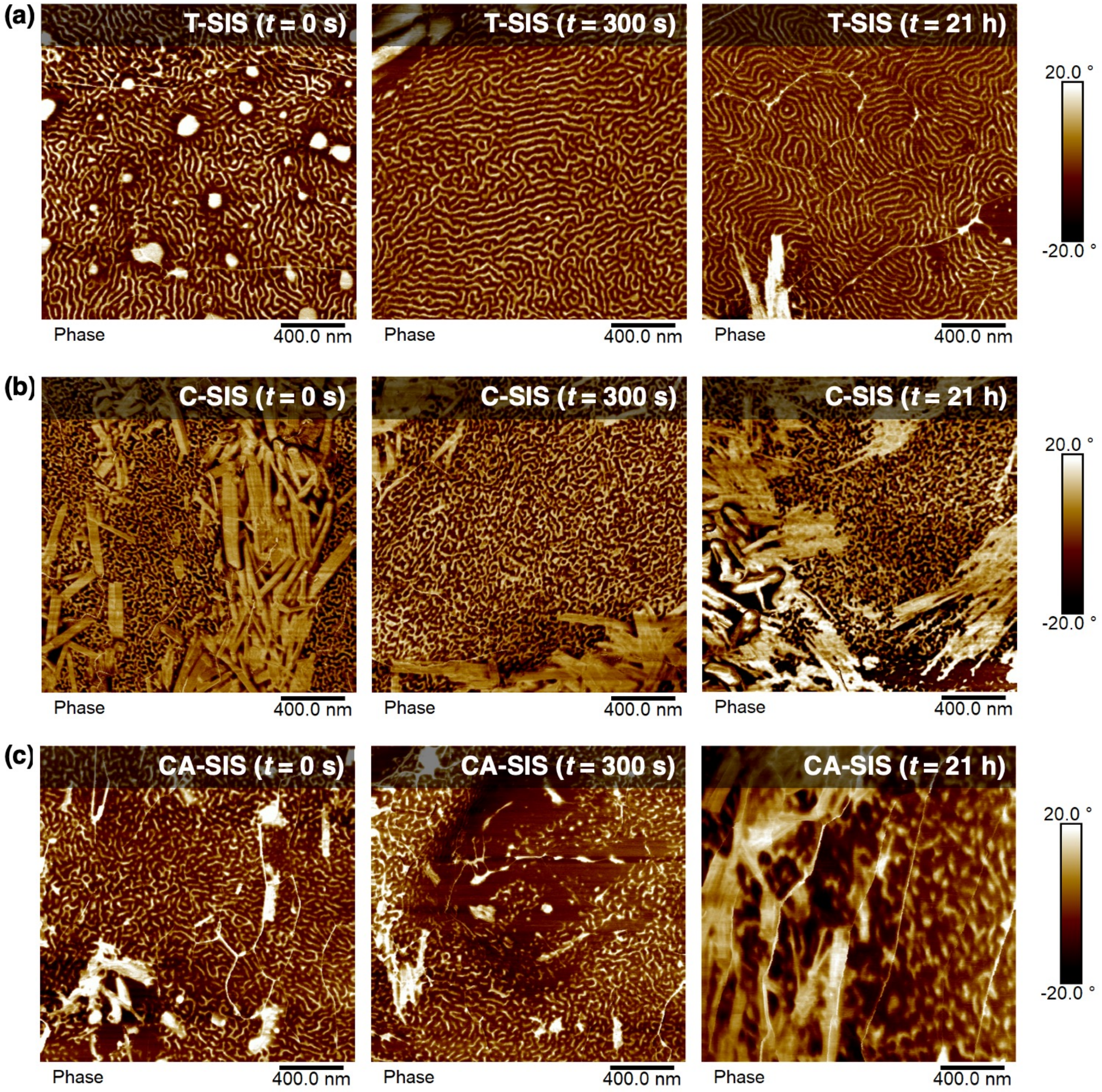

Figure S15. AFM phase images of **SIS** disks containing (a) tropolone, (b) camphor, and (c) camphoric acid before (left) and after swimming for 300 s (middle) or 21 h (right) on distilled water at 25 °C. All samples contain 0.5 wt % **PDI** dye.

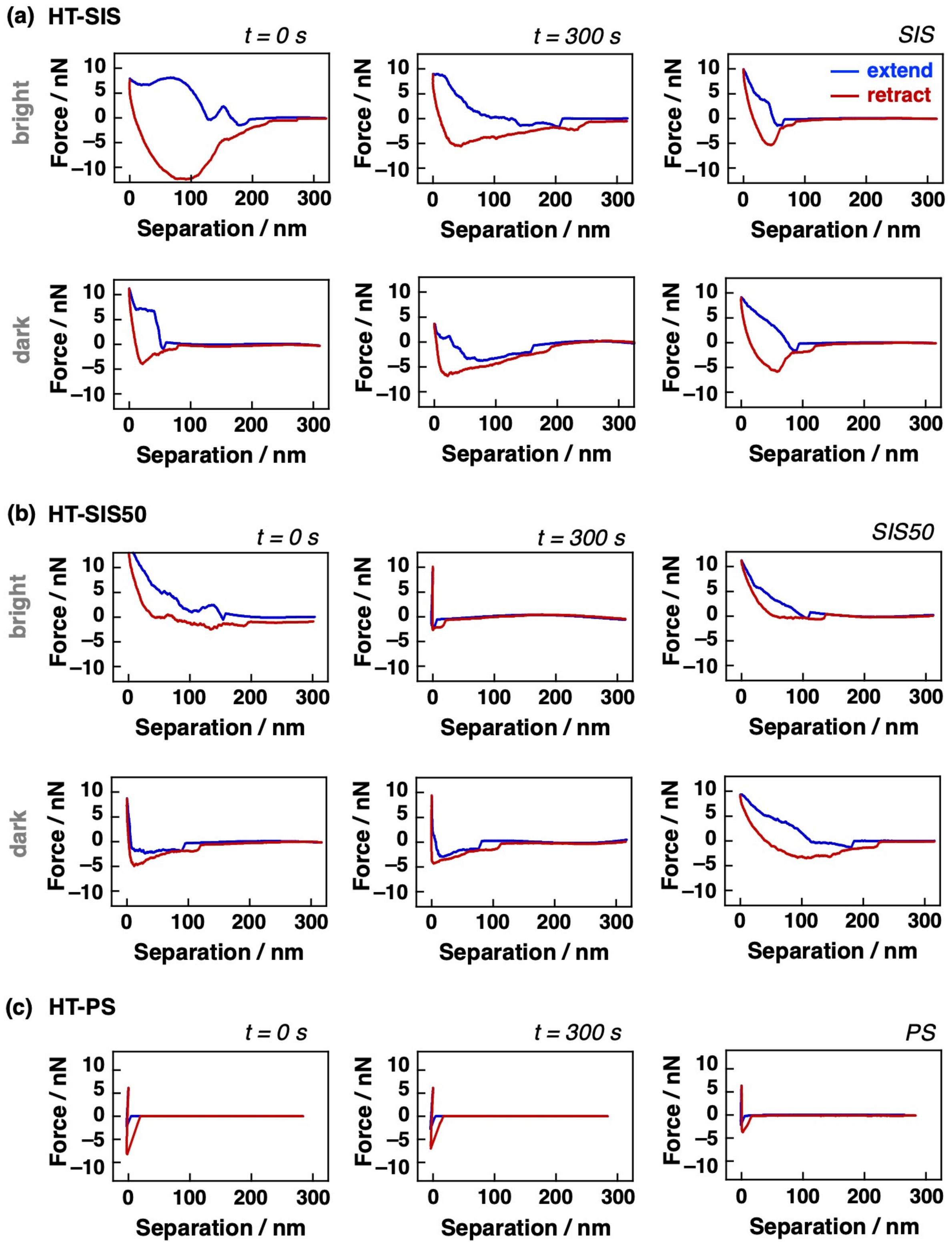

Figure S16. AFM force curves measured on the bright (top) and dark (bottom) regions of (a) **HT-SIS**, (b) **HT-SIS50**, and (c) **HT-PS** disks before (left) and after (center) swimming for 300 s on distilled water at 25 °C. Each elastomer in the absence of **HT** is also shown. Force curves for disks after 21 hours of swimming could not be reliably measured due to the surface roughness. The

hysteresis between the extending and retracting curves indicates plastic deformation characteristic of compliant materials.[S8] Assignment of the bright and dark regions of **SIS** in the presence of **HT** is not straightforward, though it was clear that at least one of the polymer blocks had hardened after swimming.[S9] Disks constructed of just high molecular weight **PS** maintained their rigidity when blended with **HT**, although **HT-PS** disks exhibited stronger adhesive forces than **PS** only disks.

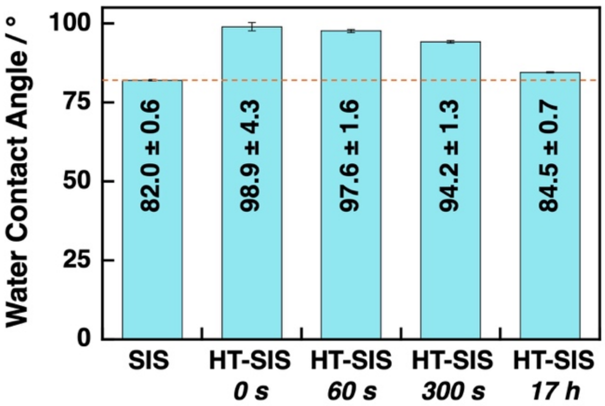

Figure S17. Average water contact angles observed for **SIS** and **HT-SIS** before and after swimming on distilled water for 60 s, 300 s, and 17 h.

Author Contributions

Atsuro Takai (A.T.) conceived and supervised the project. Lara Rae Holstein (L.R.H.) performed most of the experiments. Nobuhiko J. Suematsu (N.J.S.) conducted mathematical modeling of the self-propelled motion. L.R.H. and A.T. cowrote the original draft. All the authors reviewed and edited the manuscript.

Supporting References

(S1) Yang, Y.; Wang, Y.; Xie, Y.; Xiong, T.; Yuan, Z.; Zhang, Y.; Qian, S.; Xiao, Y. Fused perylenebisimide–carbazole: new ladder chromophores with enhanced third-order nonlinear optical activities. *Chem. Commun.* **2011**, *47*, 10749.

(S2) Shi, W.; Li, W.; Delaney, K. T.; Fredrickson, G. H.; Kramer, E. J.; Ntaras, C.; Avgeropoulos, A.; Lynd, N. A. Morphology re-entry in asymmetric PS-PI-PS' triblock copolymer and PS homopolymer blends. *J. Polym. Sci., Part B: Polym. Phys.* **2016**, *54*, 169.

(S3) Yamada, M.; Shigemune, H.; Maeda, S.; Sawada, H. Temperature and Humidity Dependence of Marangoni Convection and Its Effect on the Self-propulsion of an Oil Droplet. *Chem. Lett.* **2020**, *50*, 493.

(S4) Holstein, L. R.; Suematsu, N. J.; Takeuchi, M.; Harano, K.; Banno, T.; Takai, A. Reduction-Induced Self-Propelled Oscillatory Motion of Perylenediimides on Water. *Angew. Chem., Int. Ed.* **2024**, *63*, e202410671.

(S5) Takeuchi, H. In *Powder Technology Handbook*; 4th ed.; Higashitani, K., Makino, H., Matsusaka, S., Eds.; CRC Press: Boca Raton, US, 2019, p 171.

(S6) Nagayama, M.; Nakata, S.; Doi, Y.; Hayashima, Y. A theoretical and experimental study on the unidirectional motion of a camphor disk. *Physica D* **2004**, *194*, 151.

(S7) Tanaka, K.; Nagahiro, R.; Ohba, S.; Eishima, M. First isolation of tautomeric isomers of 4-isopropyltropolone as inclusion complex crystals. *Tetrahedron Lett.* **2001**, *42*, 925.

(S8) Chizhik, S. A.; Huang, Z.; Gorbunov, V. V.; Myshkin, N. K.; Tsukruk, V. V. Micromechanical Properties of Elastic Polymeric Materials As Probed by Scanning Force Microscopy. *Langmuir* **1998**, *14*, 2606.

(S9) Zuo, B.; Zheng, F. F.; Zhao, Y. R.; Chen, T.; Yan, Z. H.; Ni, H.; Wang, X. Stick–Slip Phenomenon in Measurements of Dynamic Contact Angles and Surface Viscoelasticity of Poly(styrene-*b*-isoprene-*b*-styrene) Triblock Copolymers. *Langmuir* **2012**, *28*, 4283.
